# Accelerated Sensitivity Analysis in High-Dimensional Stochastic Reaction Networks

**DOI:** 10.1371/journal.pone.0130825

**Published:** 2015-07-10

**Authors:** Georgios Arampatzis, Markos A. Katsoulakis, Yannis Pantazis

**Affiliations:** Dep. of Mathematics and Statistics, University of Massachusetts, Amherst, MA, United States of America; SUNY Downstate MC, UNITED STATES

## Abstract

Existing sensitivity analysis approaches are not able to handle efficiently stochastic reaction networks with a large number of parameters and species, which are typical in the modeling and simulation of complex biochemical phenomena. In this paper, a two-step strategy for parametric sensitivity analysis for such systems is proposed, exploiting advantages and synergies between two recently proposed sensitivity analysis methodologies for stochastic dynamics. The first method performs sensitivity analysis of the stochastic dynamics by means of the Fisher Information Matrix on the underlying distribution of the trajectories; the second method is a reduced-variance, finite-difference, gradient-type sensitivity approach relying on stochastic coupling techniques for variance reduction. Here we demonstrate that these two methods can be combined and deployed together by means of a new sensitivity bound which incorporates the variance of the quantity of interest as well as the Fisher Information Matrix estimated from the first method. The first step of the proposed strategy labels sensitivities using the bound and screens out the insensitive parameters in a controlled manner. In the second step of the proposed strategy, a finite-difference method is applied only for the sensitivity estimation of the (potentially) sensitive parameters that have not been screened out in the first step. Results on an epidermal growth factor network with fifty parameters and on a protein homeostasis with eighty parameters demonstrate that the proposed strategy is able to quickly discover and discard the insensitive parameters and in the remaining potentially sensitive parameters it accurately estimates the sensitivities. The new sensitivity strategy can be several times faster than current state-of-the-art approaches that test all parameters, especially in “sloppy” systems. In particular, the computational acceleration is quantified by the ratio between the total number of parameters over the number of the sensitive parameters.

## Introduction

Biological and biochemical reaction networks provide a powerful computational and modeling tool for the intrinsic understanding of fundamental mechanisms in systems biology such as metabolic, regulatory and signalling pathways. With the advent of ever-increasing computational power and the desire for more accurate representations of the physical processes at the level of the (sub-)cell or at the level of populations, larger, more complex and more sophisticated biochemical reaction networks have been developed. For instance, reconstruction for genome-scale, steady-state models of metabolic networks, macromolecular synthesis and multiscale systems biology necessitates reaction networks with (tens of) thousands of reactions [1–3]. The enormous increase in the size of modeled reaction networks presents severe modeling and computational challenges, among others the understanding, designing, inferring and predicting the properties and the behavior of the output, especially when the stochasticity is a prerequisite for correct modeling, e.g. [4, 5]. Indeed, for low species populations, stochastic models are critical for the correct representation of the inherent randomness and discrete nature of intracellular networks. In particular, a ubiquitous problem in biochemical reaction networks is to quantify the response of the system when perturbations on the input or on the parameters of the system are performed. Subsequently, questions on the robustness, (structural) identifiability, experimental design, uncertainty quantification, estimation and control can be addressed, [6]. The quantification of system response to parameter perturbations is called sensitivity analysis and is an indispensable analysis tool for the study of kinetic models [6, 7].

For large-scale reaction networks, sensitivity analysis is especially important due to the confluence of the nonlinear, stochastic and typically non-equilibrium statistical mechanics characteristics of the models. Additionally, the large number of parameters yields a very high number of sensitivity indices needed to be estimated, increasing by orders of magnitude the overall computational cost when compared to simply the forward simulation of the model. Finally, the stochasticity may also result in high variance in the estimators of the sensitivity indices, adding both further computational cost and uncertainty in the predictions of the sensitivity analysis. This paper addresses precisely such challenges, namely the parametric sensitivity analysis of high-dimensional stochastic reaction networks, both in the size of the parameter vector (large parameter space) and the number of species (large state space).

Recently, there has been significant progress in developing sensitivity analysis tools for low-dimensional stochastic dynamics, modeling well-mixed chemical reactions and biological networks. Some of the mathematical tools includes log-likelihood methods and Girsanov transformations [8–10], polynomial chaos expansions [11], finite difference methods and their variants [12–14], as well as pathwise sensitivity methods [15]. Moreover, in recent years there has been a significant development in sensitivity analysis software for reaction networks. Existing modeling software like COPASI [16], PottersWheel [17], SensSB [18] analyzes parameter sensitivity with deterministic modeling of dynamical systems. Sensitivity analysis for stochastic reaction networks has been implemented in software packages like SPSens [19], StochSS [20], StochSens [21] and ISAP [22].

In another and complementary direction, recent sensitivity analysis approaches have been proposed as means to quantify the overall behavior of the reaction network and not just the response of a specific observable function. These sensitivity analysis methods employ information theory metrics such as the relative entropy (also known as Kullback-Leibler divergence) as well as the Fisher Information Matrix (FIM). Moreover, taking into account the fact that the knowledge of the stationary distribution is rarely known in biochemical reaction networks, these information-based methods resort either on linearized Gaussian approximations of the underlying process [23] or they rely on path-space distribution calculations, [24, 25]. The latter approach is exact since no approximation is necessary, while it is gradient-free in the sense that a single model (parameter) simulation is carried out, resulting in reduced-variance estimators. Overall, gradient-free sensitivity analysis methods such as the ones proposed in [23–25] are highly appropriate for systems with a high-dimensional parameter space since they allow for an efficient exploration of the parameter space without the calculation of a very high number of (directional) derivatives.

However, existing sensitivity analysis methods are not capable of estimating the sensitivity analysis of specific quantities of interest (observables) for large stochastic networks, in a computationally efficient and accurate manner. Indeed, beginning with the deterministic models, their sensitivity analysis is fairly easily formulated by the adjunct system of differential equations which is the ODE system that governs the dynamics of the reaction network augmented by the ODEs for the derivatives with respect to the parameters [7]. However, this is not always adequate because such models do not take into account the intrinsic stochasticity which crucially affects the behavior of the system [4, 5]. On the other hand, stochastic sensitivity analysis methods based on information criteria [23, 24] are also inexpensive in terms of computational cost since they can handle large networks with many parameters due to their gradient-free nature, [25], however, they are not observable-based, hence may not provide precise analysis for specific quantities of interest. Finally, observable-based approaches such as finite-difference (gradient) methods can have an overwhelming computational cost, either due to high variance in the gradient estimators [26] or the high-dimensional state space [14]; in the latter case such computations can be prohibitively expensive when they need to be run over and over again for many parameter perturbations related to the sensitivity indices.

Here we demonstrate that the aforementioned methods can be combined and deployed together by means of a new sensitivity bound which incorporates the variance of the quantity of interest as well as the FIM, see inequality (4). The proposed strategy is a two-step hierarchical approach where in the first step the insensitive observables and parameters are found and eliminated from further analysis with controlled accuracy; the second step targets the remaining (potentially) sensitive parameters and observables:

- *Step 1*: Screen the parameters through a computational inexpensive labelling of the insensitive parameters based on a sensitivity bound (SB) derived from a path-space Cramer-Rao inequality (see (Eq 4)). The sensitivity index (SI) for an observable (see (Eq 3) for a definition) can be bounded by the square root of the variance of the observable multiplied by the diagonal FIM elements. Here we utilize the pathwise FIM [25] which quantifies information from both the steady states and the dynamics of the reaction network.

To this end note that since the SB is an upper bound of the SIs (see (Eq 4)), neither guarantees that large values of the bound imply large SIs, nor infers information on their order. Therefore, for the SIs where the SB is large, a more accurate sensitivity analysis method is needed. Indeed, in the second step of the strategy, an observable-based sensitivity analysis algorithms is applied to the potentially sensitive parameters and observables:

- *Step 2*: Employ an estimator for the SIs on the remaining potentially sensitive parameters. Here we choose the gradient estimator given by the coupling method [13] which is a finite-difference approach with reduced variance, even in high-dimensional systems, [14].

The pathwise FIM quantifies the information change of the path-space distribution (i.e., the distribution of the species trajectories) of stochastic processes under perturbations, [25]. Furthermore, the estimation of the pathwise FIM is very efficient because a single model simulation is required for the computation of the whole matrix, while the variance of the statistical estimator is typically small. The acceleration in sensitivity analysis due to the proposed strategy can be very significant especially when sloppy systems are considered, [25, 27, 28], and most of the parameters are expected to be screened out as insensitive from *Step 1*. Moreover, the proposed strategy offers a simple way to rationally balance accuracy and computational cost, selecting the number of insensitive parameters that need to be discarded. The discrimination between sensitive and insensitive parameters can be performed by a user-determined tolerance or by the availability of computational resources. The proposed strategy, through the SB, guarantees that the SIs for the insensitive parameters will lie below the value of the tolerance.

A detailed demonstration and validation of the proposed strategy applied on three biological models is provided. The p53 model [29–31] is presented as a small-size example, having 21 SIs, introducing the idea of screening out negligible sensitivities. Due to the nontrivial stationary behavior of the p53 model, with random and persistent oscillations of the solution, sensitivities of observables such as the amplitude and the frequency of the oscillations are explored. A more complex model with a large number of species and parameters, the Epidermal Growth Factor Receptor (EGFR) model [32–34], is studied next. The EGFR model is studied both in the transient and in the stationary regimes allowing us to demonstrate the applicability of the proposed strategy in both settings. In this particular example, more than half of the sensitivities can be excluded by the computationally inexpensive SB of *Step 1*. Finally, a protein homeostasis model, [35], with a total of 4160 SIs is presented as a “sloppy” example, where more that 85% of the total SIs can be safely ignored having a guaranteed maximum bound by the screening in *Step 1*. The results of the proposed strategy are compared against the results of the full coupling method applied to all SIs without any screening. The computational cost of the two methods, measured in number of samples, is compared and found that the two-step strategy can accelerate up to approximately $\frac{K}{K^{\prime }}$ times the sensitivity analysis where *K* is the total number of parameter, while *K*′ is the number of parameters remaining after the screening in *Step 1*; as we show in the demonstrated examples, the SB calculation in *Step 1* is negligible since it is at least one order of magnitude less expensive compared to a single run of the coupling method in *Step 2*.

The paper is organized as follows. In the Methods section, the sensitivity analysis strategy is presented. The validation of the proposed sensitivity analysis approach is provided in the Results section, where one small and two large biochemical reaction networks are tested while the computational advantages are quantified and presented in the Discussion section.

## Methods

A well-mixed reaction network with *N* species, **S** = {*S*_1_, …, *S*_*N*_}, and *J* reactions, **R** = {*R*_1_, …, *R*_*J*_} is considered. The state of the system at any time *t* ≥ 0 is denoted by an *N*-dimensional vector **X**_*t*_ = [*X*_*t*,1_, …, *X*_*t*, *N*_]^*T*^ where *X*_*t*, *i*_ is the number of molecules of species *S*_*i*_ at time *t*. Let the *N*-dimensional vector ν_j_ correspond to the stoichiometry vector of *j*-th reaction such that *ν*_*i*, *j*_ is the stoichiometric coefficient of species *S*_*i*_ in reaction *R*_*j*_. Given that the reaction network at time *t* is in state **X**_*t*_ = **x**, the propensity function, $a_{j}^{\theta }(\text{x})$, is defined so that the infinitesimal quantity $a_{j}^{\theta }(\text{x})dt$ gives the probability of the *j*-th reaction to occur in the time interval [*t*, *t* + *dt*]. Propensities are typically dependent on the state, **x**, of the system and the reaction conditions of the network which are made explicit by the parameter vector *θ* ∈ ℝ^*K*^. Mathematically, {**X**_*t*_}_*t* ∈ ℝ_+__ is a continuous-time Markov chain (CTMC) with countable state space *E* ⊂ ℕ^*N*^. The transition rates of the CTMC are the propensity functions $a_{j}^{\theta }(\cdot ),j=1,\ldots ,J$. The transition rates determine the clock of the updates (or jumps) from a current state **x** to a new (random) state $x^{\prime }=x+\nu_{j}$ through the total rate $a_{0}^{\theta }(\text{x}):=\sum_{j=1}^{J}a_{j}^{\theta }(\text{x})$ while the transition probabilities of the process are determined by the ratio $\frac{a_{j}^{\theta }(\text{x})}{a_{0}^{\theta }(\text{x})}$. In order to have a complete description of the reaction network, an initial distribution of the state at time instant *t* = 0 denoted by *ν* ^*θ*^(·) is also needed.

There are exact algorithms for the simulation of the reaction network such as the stochastic simulation algorithm (SSA) of Gillespie [36, 37] or the next reaction algorithm of Gibson and Bruck [38] or the constant-time kMC algorithm of Slepoy et al. [39] as well as inexact approximation algorithms such as *τ*-leap [40] and several variations of it [41, 42]. As an example, given that the system is at the state **X**_*t*_ = **x** at time *t*, SSA computes the waiting time *δt* as a random number drawn from an exponential distribution with the total rate $a_{0}^{\theta }(\text{x})$, while the *R*_j*_ reaction occurs where j* ∈ {1, …, *J*} is chosen such that $\sum_{j=1}^{j^{*}-1}a_{j}^{\theta }(\text{x})<ua_{0}^{\theta }(\text{x})<\sum_{j=j^{*}}^{J}a_{j}^{\theta }(\text{x})$ and *u* is a random number uniformly chosen in the interval [0, 1]. The new state is given by **X**(*t* + *δt*) = **x**′ = **x** + *v*_j*_.

The path space distribution of the stochastic process on the time-interval [0, *T*] is denoted by $Q_{\text{[0,T]}}^{\theta }$. Notice that the dependence of the path space distribution to the initial distribution is made implicit for notational simplicity. Intuitively, the path space is the set of all possible trajectories in [0, *T*], generated by SSA for the particular reaction network while the path space distribution is the probability to observe a particular trajectory. For a concrete and simple example of a path distribution, we refer to Section 2 in S1 File where Discrete-Time Markov Chains (DTMCs) are considered. As we shall show next (see also S1 File), the path space perspective is easy to implement exploiting concepts from information theory and from non-equilibrium statistical mechanics.

We now turn our attention to observables of the stochastic process, **X**_*t*_. We denote by **F**(·) = [*F*_1_(·), …, *F*_*L*_(·)]^*T*^ the vector with *L* state-dependent observable functions, *F*_ℓ_: 𝓧 → ℝ, ℓ = 1, …, *L*. Two typical options for the observable function are the time-average of a function as well as the value of a function at a specific time instant. The time-average observable is defined in a general setting as

$$F_{\ell }\left({\left\{X_{t}\right\}}_{0}^{T}\right)=\frac{1}{T}\int_{t=0}^{T}f_{\ell }\left(X_{t}\right)dt\;,$$ (1)

while the time-specific observable is defined as

$$F_{l}\left({\left\{X_{t}\right\}}_{t=0}^{T}\right)=f_{l}\left(X_{T}\right)\;.$$ (2)

The most common observable is the population of the ℓ-th species, i.e., the projection of the state vector to the ℓ-th direction (*f*_ℓ_(**x**) = *x*_ℓ_), however, other observable functions such as correlations between various species of the network, time-correlations for a specific species as well as switching or exit times can be considered. Another important discrimination for the observable functions stems from the time regime where the stochastic process is sampled. There are two important regimes; the stationary regime where the process is at equilibrium and the transient regime where the stochastic process initialized far from equilibrium and in the course of time it relaxes towards the steady states. At the stationary regime, the initial distribution of the stochastic process is the stationary distribution and both time-average and time-specific observables produce the same ensemble averages since $E_{Q_{\text{[0,T]}}^{\theta }}[\frac{1}{T}\int f{\text{(X}}_{\text{t}})dt]=E_{Q_{\text{[0,T]}}^{\theta }}{[f(\text{X}}_{\text{T}}\text{)]}=E_{\mu^{\theta }}[f(\text{x})\text{]}$ where *μ* ^*θ*^: *E* → ℝ is the stationary distribution while $E_{P}[f]$ denotes the expectation of *f*(·) with respect to the probability *P* (i.e., $E_{P}[f(x)]:=\int f(x)P(x)dx$). Moreover, due to the (assumed) ergodicity property of the reaction network, it is typical to obtain ensemble averages and statistics from time-averaged observables since ergodicity asserts that ${\text{lim}}_{T\to \infty }\frac{1}{T}\int f({\text{X}}_{t})dt=E_{\mu^{\theta }}[f(\text{x})]$.

The goal of this paper is to describe an efficient and highly resolved strategy to compute the parameter sensitivities on the observable functions, i.e., to compute the sensitivity matrix, *S* ∈ ℝ^*K* × *L*^, defined element-wise by

$$S_{k,\ell }=\frac{\partial }{\partial \theta_{k}}E_{Q_{\left[0,T\right]}^{\theta }}\left[F_{\ell }\left({\left\{X_{t}\right\}}_{t=0}^{T}\right)\right]\;,\;\;\;k=1,\ldots ,K\;\&\;\ell =1,\ldots ,L\;.$$ (3)

The element, *S*_*k*, ℓ_, is the Sensitivity Index (SI) of the ℓ-th observable to the *k*-th parameter. The proposed strategy is separated into two steps where, in the first step, a Sensitivity Bound (SB) is computed for each SI. The evaluation of the SB is based on tools from estimation theory [43, 44] and information theory [45]. The SB is a product of two factors where the first one depends on the properties of the observable function while the second factor depends only on the properties of the underlying path space distribution of the stochastic process (see (Eq 4) and (Eq 6) below). The computational efficiency of the SB stems from its factorization into two terms each one quantifying different aspects of the SIs. Then, in the second step, we apply a computationally more expensive but accurate sensitivity estimation method. In particular, we use the coupling method [13], however, applied only on the potentially sensitive SIs since from the first step the least sensitive SIs have been screened out with a controlled error given by the SB. We discuss these two components of our proposed methodology next.

### Step 1: Screening out insensitive parameters and observables

In parameter estimation theory, the Cramer-Rao theorem states that the variance of an estimator cannot be smaller than the inverse of the FIM [43, 44]. Here, we are not interested to bound the variance of the estimator from below as in the Cramer-Rao theorem, but rather to bound the bias of the estimator from above which in our context corresponds to the SI. Thus, the sensitivity bound (SB) can be obtained by rearranging the generalized Cramer-Rao bound for a biased estimator, [43, 44]. In particular, when the distribution in the Cramer-Rao theorem is the path distribution, the absolute SI of the ℓ-th observable is bounded by the inequality

$$\left|S_{k,\ell }\right|\leq B_{k,\ell }:=\sqrt{{\text{Var}}_{Q_{\left[0,T\right]}^{\theta }}\left(F_{\ell }\right)}\sqrt{I{\left(Q_{\left[0,T\right]}^{\theta }\right)}_{k,k}}\;,$$ (4)

where $I{\text{(Q}}_{\text{[0,T]}}^{\theta }\text{)}$ is the *K* × *K* pathwise FIM. We also recall that the path space distribution of the stochastic process on the time-interval [0, *T*] is denoted by $Q_{\left[0,T\right]}^{\theta }$. This inequality is a general SB which, assuming that the estimation of the variance of the observable and the pathwise FIM is tractable and fast, can be utilized to discard the most insensitive SIs. Indeed, if the right hand side of the inequality is small then the corresponding SI is also small. In other words, given a specific observable, diagonal FIM elements with small values imply low SIs. However, notice that large FIM values in (Eq 4) do not necessarily imply large SIs or any information on ranking the SIs with high values. From this latter observation stems the need for the second step in the proposed sensitivity analysis strategy.

Overall, with the cost of estimating an upper bound instead of the actual values, the estimation of *K* × *L* sensitivity indices is reduced to the estimation of *L* variances and *K* elements of the FIM which is a significant reduction especially when the studied system is high-dimensional both in the parameter space (*K* ≫ 1) and in state space (*N* ≫ 1). In typical cases, *F*_ℓ_(**x**) = *x*_ℓ_ (i.e., projection operators as observables) hence *L* = *N* ≫ 1, however, when correlations between species are of interest then *L* = *N*(*N* − 1)/2 and the computation of the sensitivity matrix (Eq 3) becomes readily intractable.

From an information theory perspective, pathwise FIM is the Hessian of the pathwise relative entropy which geometrically corresponds to the curvature around its minimum value [24]. For a definition of pathwise relative entropy as well as its properties for both discrete-time Markov chain (DTMC) and CTMC cases we refer to S1 File. The SB (Eq (4)) can be also derived as a limit of a variational inequality which relates the weak error between two path distributions with their pathwise relative entropy (see S1 File for details). Another derivation of the SB can be obtained as a limit of the Chapman-Robbins inequality which incorporates the chi-squared divergence [46].

We also remark that (Eq 4) can be generalized to provide a SB for any combination of the parameters (i.e., bound the directional derivative). The estimation of pair directional derivatives provides a manner to infer information about correlations between parameters and how combinations of perturbation affect the output of the reaction network. Notice that the order of the size of pair directional derivatives is *O*(*K* ^2^) and for each pair a new call of the gradient estimation is required making the computational cost of estimating the correlations intractable in high-dimensional systems. However, using the proposed strategy in combination with the following sensitivity bound the estimation of correlations between parameters is tractable. Mathematically, for any *v* ∈ ℝ^*K*^ with ∣*v*∣ = 1 and denoting the directional derivative by $\partial_{v}E_{Q_{\left[0,T\right]}^{\theta }}[F]$, it holds that the sensitivity bound for an observable function *F*(·) at the direction *v* is given by

$$\left|\partial_{v}E_{Q_{\left[0,T\right]}^{\theta }}\left[F\right]\right|\leq \sqrt{{\text{Var}}_{Q_{\left[0,T\right]}^{\theta }}\left(F\right)}\sqrt{v^{T}I\left(Q_{\left[0,T\right]}^{\theta }\right)v}.$$ (5)

It was shown (Fig. 1 in [25]) that pathwise FIM has a block-diagonal structure which reduces the non-zero elements of the matrix from *O*(*K* ^2^) to *O*(*K*) thus reducing the number of elements needed to be estimated. Furthermore, inequality (Eq 5) combined with direct spectral analysis of the block-diagonal pathwise FIM can infer the least sensitive directions of the system as well as the most sensitive candidate directions [23, 25].

The computation of the pathwise FIM, $I(Q_{\left[0,T\right]}^{\theta })$, necessitates the explicit knowledge of the probability function which is not always possible. However, in the setting of Markov processes, explicit formulas for the pathwise FIM exist [25]. Indeed, using the properties for the pathwise relative entropy, we are able to derive explicit formulas for the pathwise FIM. Next, we provide such formulas for both the stationary and the transient regime. Note that, as expected, the SB is time-independent in the stationary regime.

#### Stationary regime

In stationary regimes of stochastic processes, the probability law of the process is time-independent. Therefore, the typical observables utilized in steady state regimes are the time-averaged observables given by (Eq 1). Then, it can be shown for the stationary regime and time-averaged observables that the SB given by (Eq 4) becomes time-independent and it can be rewritten as

$$|S_{k,\ell }|\leq \sqrt{\tau_{\mu^{\theta }}\left(f_{\ell }\right)}\sqrt{I_{H}{\left(Q^{\theta }\right)}_{k,k}}\;,$$ (6)

where $\tau_{\mu^{\theta }}(f_{\ell })$ is the Integrated Autocorrelation Time (IAT) while 𝓘_ℋ_(*Q* ^*θ*^) is the FIM of the so called Relative Entropy Rate [24, 25] (for a proof, see Section 1.1 of S1 File). The IAT is given by (see Section 3.2, S1 File for a derivation)

$$\tau_{\mu^{\theta }}\left(f\right)=\int_{-\infty }^{\infty }<f\left(X_{t}\right)-E_{\mu^{\theta }}\left[f\right],f\left(X_{0}\right)-E_{\mu^{\theta }}\left[f\right]{>}_{\mu^{\theta }}dt$$ (7)

where $<f(X_{t})-E_{\mu^{\theta }}[f(x)],f(X_{0})-E_{\mu^{\theta }}[f(x)]{>}_{\mu^{\theta }}$ is the stationary covariance between *f*(**X**_*t*_) and *f*(**X**_0_). We remark that IAT has been used in a series of problems in probability and statistics to measure the performance of samplers and estimators, [47]. Many of the state-of-the-art estimators of IAT give non-reliable results while those that can give satisfactory results depend on parameters that need to be tuned by visual inspection of the autocorrelation time. In fact, we have found that the most reliable estimator of IAT is the one presented in S2 File.

In the context of well-mixed reaction networks and assuming smoothness of the propensity functions, $a_{j}^{\theta }(\text{x}),j=1,\ldots ,J$, with respect to the parameter vector, *θ*, the pathwise FIM is explicitly written as, [25],

$$I_{H}\left(Q^{\theta }\right)=E_{\mu^{\theta }}[\sum_{j=1}^{J}a_{j}^{\theta }\left(x\right)\nabla_{\theta }\log a_{j}^{\theta }\left(x\right)\nabla_{\theta }\log a_{j}^{\theta }{\left(x\right)}^{T}]\;.$$ (8)

Thus, the time-independent pathwise FIM can be practically estimated as an ergodic average. Statistical estimators for the stationary pathwise FIM are provided in S2 File.

*Remark*: As a concrete example of (Eq 6) consider a stochastic process whose autocorrelation function with respect to an observable *f* decays exponentially fast with rate (i.e., decorrelation time) *τ*_*d*_ = *τ*_*d*_(*f*, *μ* ^*θ*^). Then, the IAT satisfies $\tau_{\mu^{\theta }}(f)=2\tau_{d}{\text{Var}}_{\mu^{\theta }}(f)$, [47], and then the stationary SB (Eq 6) becomes

$$|S_{k,\ell }|\leq \sqrt{2\tau_{d}}\sqrt{{\text{Var}}_{\mu^{\theta }}\left(f_{\ell }\right)}\sqrt{I_{H}{\left(Q^{\theta }\right)}_{k,k}}\;.$$ (9)

#### Transient regime

In the transient regime, the sensitivity bound is given by the general inequality (Eq 4). Nevertheless, in terms of implementation, the estimators of the transient regime are the same as in the stationary regime. This is evident in (Eq 10) below, which is derived in Section 3.1, S1 File.

More precisely, assuming that the propensity functions are differentiable with respect to the parameter vector *θ*, the pathwise FIM for the transient regime is given by

$$I\left(Q_{\left[0,T\right]}^{\theta }\right)=I\left(\nu^{\theta }\right)+\int_{0}^{T}I_{H}\left(Q_{t}^{\theta }\right)dt\;,$$ (10)

where 𝓘(*ν* ^*θ*^) is the FIM of the initial distribution, *ν* ^*θ*^, and **X**_*t*−_ denotes the the left-side limit at time *t*. The process $I_{ℋ}(Q_{t}^{\theta })$ can be viewed as the instantaneous pathwise FIM given by

$$I_{H}\left(Q_{t}^{\theta }\right)=E_{Q_{\left[0,t\right]}^{\theta }}[\sum_{j=1}^{J}a_{j}^{\theta }\left(X_{t-}\right)\nabla_{\theta }\log a_{j}^{\theta }\left(X_{t-}\right)\nabla_{\theta }\log a_{j}^{\theta }{\left(X_{t-}\right)}^{T}]\;,$$ (11)

which readily reduces to (Eq 8), in the stationary regime. The formula of the instantaneous pathwise FIM is not as simple as in the DTMC case (see Section 2.1, S1 File) because the waiting time of the jumps is now random, however, the statistical estimator for the pathwise FIM in the transient regime is as simple as in the stationary regime. In fact it has exactly the same formula as we show in S2 File. Finally, we would like to point out that a software package called ISAP has been recently launched [22] that computes among other quantities the pathwise FIM in both transient and stationary regimes further strengthening the usefulness of the proposed strategy.

### Step 2: Finding and ranking the most sensitive SIs

In this second step of the proposed strategy, we employ a computationally more expensive but accurate sensitivity estimation method: we use the the coupling method [13], which is only applied on the potentially sensitive SIs since from the *Step 1* the least sensitive SIs have been screened out with a controlled error given by the SB. We discuss the coupling methodology next.

First, the SI defined in (Eq 3) is approximated by a second-order finite difference scheme as

$$S_{k,\ell }\approx {\tilde{S}}_{k,\ell }=\frac{1}{2\epsilon_{0}}(E[F_{\ell }\left(X^{+}\right)]-E[F_{\ell }\left(X^{-}\right)])\;,$$ (12)

where we use the abbreviated notation $X^{\pm }={\left\{X_{t}^{\theta_{k}\pm \epsilon_{0}}\right\}}_{t=0}^{T}$ while *ϵ*_0_ ∈ ℝ and *ϵ*_0_ ≪ 1. In this study we set *ϵ*_0_ = 0.1. Notice also that, for notational simplicity, we dropped the dependence on the underlying path space distribution from the expectation. The variance of the estimator in (Eq 12) is proportional to

$$\begin{array}{cc} \mathrm{Var}[F_{\ell }\left(X^{+}\right)-F_{\ell }\left(X^{-}\right)] & =\mathrm{Var}[F_{\ell }^{2}\left(X^{+}\right)]+\mathrm{Var}[F_{\ell }^{2}\left(X^{-}\right)]-\mathrm{Cov}(F_{\ell }\left(X^{+}\right),F_{\ell }\left(X^{-}\right))\;. \end{array}$$ (13)

In order to minimize the variance of the estimator we have to correlate the processes in a way such that the covariance $\text{Cov}(F_{\ell }(X^{+}),F_{\ell }(X^{-}))$ is maximized since the first two terms in (Eq 13) do not depend on the correlation between the two processes, **X** ^+^ and **X** ^−^. One way to correlate these two processes is the stochastic coupling method, [13, 14], where it has been proved that the coupling between the two processes indeed reduces the variance of the estimator of (Eq 12). S3 File contains implementation details regarding the coupling method. Notice also that the coupling method is employed *K* times; one for each parameter, *θ*_*k*_, *k* = 1, …, *K* and if pair directional derivatives needed to be computed then the coupling method must be invoked *K*(*K*−1)/2 times which for networks with high-dimensional parameter space is prohibitively expensive.

**Fig 1:**
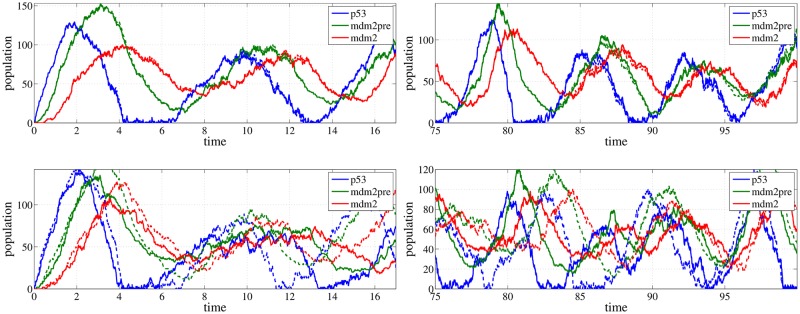
Trajectories of the species of the p53 model. The solid and the dashed lines correspond to the unperturbed and the perturbed parameters respectively. Left panel: The result of the coupled algorithm (upper plot) in comparison to the result of two completely uncoupled runs (lower plot) is presented. Note that the coupled algorithm produces correlated paths, that are close to each other, thus leading to smaller variance in the estimator (12). Right panel: The same computation as in the left panel for a larger time interval. The coupling method manages to keep the trajectories very close.

In Fig 1, the trajectories of the species of the p53 model (details in Results below) obtained from the coupling method are compared with two completely uncoupled trajectories. Even when the processes have random oscillations, the coupling method manages to keep the trajectories very close. As it is also evident from Fig 1 this is also true even for long times. On the other hand, the uncoupled trajectories start to separate shortly after their starting point and for longer times the peaks of the oscillatory trajectories are in completely different positions as it can be seen in the right lower plot of Fig 1.

### How to use the proposed strategy

In this subsection, we describe some of the ways the proposed methodology can be used in practice, but clearly other approaches are also possible.

#### Setting a maximum number of SIs

In this first perspective, we assume that the user is given a computational resources budget that allows the simulation of at most *M* sensitivity indices. Two important questions arise:

(a) Which SIs, *S*_*k*, ℓ_, should be chosen?
(b) Are there any guarantees regarding the magnitude of the remaining SIs?

In order to answer these questions we sort the SBs, see (Eq 4), in a descending order and define the set

$$C_{M}:=\{\text{all}\;\left(k,\ell \right):\;B_{k,\ell }\;\text{is}\;\text{one}\;\text{of}\;\text{the}\;M\;\text{largest}\;\text{SBs}\}.$$ (14)

Let also

$$\mathrm{TOL}\left(M\right):=\max_{\left(k,\ell \right)\notin C_{M}}B_{k,\ell }.$$ (15)

Then the answers to the above questions are: (a) Choose all pairs (*k*, ℓ) ∈ *C*_*M*_, (b) for all (*k*, ℓ) ∉ *C*_*M*_ we have from the inequality (4) that,

$$|S_{k,\ell }|\leq B_{k,\ell }\leq \mathrm{TOL}\left(M\right).$$ (16)

Thus, the proposed strategy guarantees that the discarded SIs will be less or equal than TOL(*M*). Note that inequality (Eq 16) quantifies the error in the proposed methodology.

#### Setting a pre-specified tolerance

On the other hand, a user can also take advantage of some pre-existing intuition regarding the modeled system, to argue that under a pre-specified tolerance the SIs can be discarded as insensitive. In this case we define the set,

$$C_{\mathrm{TOL}}:=\{\text{all}\;\left(k,\ell \right):\;B_{k,\ell }\geq \mathrm{TOL}\;\},$$ (17)

where TOL is the pre-specified tolerance. As in the previous case, where the maximum number of SIs to be computed is fixed, the user will employ a gradient estimation method (here a finite difference coupled algorithm is proposed, see Eq (12)) to compute the SIs, *S*_*k*, ℓ_, for all pairs (*k*, ℓ) in $C_{\text{TOL}}$ with the guarantee that the discarded SIs will have magnitude less than TOL.

## Results

In this section, we present and validate the proposed sensitivity analysis strategy in three biological reaction networks. The first example is the p53 model which is a reaction network with five reactions, three species and seven parameters. It is a small but interesting system due to the nontrivial long-time dynamics exhibiting random oscillations. Here, p53 is used as an introductory example to present and test the proposed strategy. Then, we present and validate the proposed strategy for the Epidermal Growth Factor Receptor (EGFR) model in the transient as well as in the stationary regime showing that our method can be equally applied at both regimes. Finally, we discuss a protein homeostasis model with a total number of 4160 SIs. This is a large-scale realistic model with sloppy characteristics.

The comparison of computational costs between the proposed strategy and the direct calculation of all SIs is discussed separately, both in a general context as well as concretely for the two latter examples; we refer to the Discussion section below.

### A p53 model

The p53 gene plays a crucial role for effective tumor suppression in humans as its universal inactivation in cancer cells suggests [29–31]. The p53 gene is activated in response to DNA damage and gives rise to a negative feedback loop with the oncogene protein Mdm2. Models of negative feedback are capable of oscillatory behavior with a phase shift between the gene concentrations. Here, we validate the proposed sensitivity analysis strategy to a simplified reaction network between three species, p53, Mdm2-precursor and Mdm2 introduced in [31]. The model consists of five reactions and seven parameters provided in Tables 1 and 2. The nonlinear feedback regulator of p53 through Mdm2 takes place in the second reaction while the remaining four reactions fall in the mass action kinetics category. Due to these mechanisms a nontrivial steady state regime characterized by random oscillations.

**Table 1:** The reaction table where *x* corresponds to p53, *y*_0_ to Mdm2-precursor while *y* corresponds to Mdm2. The state of the reaction model is defined as **x** = [*y*, *y*_0_, *x*]^*T*^ while the parameter vector is defined as *θ* = [*b*_*x*_, *a*_*x*_, *a*_*k*_, *k*, *b*_*y*_, *a*_0_, *a*_*y*_]^*T*^.

Event	Reaction	Rate	Rate’s derivative
*R*_1_	∅ → *x*	*a*_1_(**x**) = *b*_*x*_	∇_*θ*_ *a*_1_(**x**) = [1, 0, 0, 0, 0, 0, 0]^*T*^
*R*_2_	*x* → ∅	$a_{2}(\text{x})=a_{x}x+\frac{a_{k}y}{x+k}x$	∇_*θ*_ *a*_2_(**x**) = [0, *x*, *xy*/(*x* + *k*), −*a*_*k*_ *xy*/(*x* + *k*)^2^, 0, 0, 0]^*T*^
*R*_3_	*x* → *x* + *y*_0_	*a*_3_(**x**) = *b*_*y*_ *x*	∇_*θ*_ *a*_3_(**x**) = [0, 0, 0, 0, *x*, 0, 0]^*T*^
*R*_4_	*y*_0_ → *y*	*a*_4_(**x**) = *a*_0_ *y*_0_	∇_*θ*_ *a*_4_(**x**) = [0, 0, 0, 0, 0, *y*_0_,0]^*T*^
*R*_5_	*y* → ∅	*a*_5_(**x**) = *a*_*y*_ *y*	∇_*θ*_ *a*_5_(**x**) = [0, 0, 0, 0, 0, 0, *y*]^*T*^

**Table 2:** Parameter values for the p53 model.

Parameter	*b*_*x*_	*a*_*x*_	*a*_*k*_	*k*	*b*_*y*_	*a*_0_	*a*_*y*_
Value	90	0.002	1.7	0.01	1.1	0.8	0.8

Since the demonstrated model admits persistent, random oscillations we choose as observable the amplitude of the oscillations for each of the three species. We extract the value of this observable from the Power Spectral Density (PSD) [48] of the species time-series which is defined as the Fourier transform of the autocorrelation function of the species time-series. The PSD of a continuous-time process, *X*_*t*_, denoted by $|\hat{X}{|}^{2}$, can be also given by

$$|\hat{X}{|}^{2}\left(\xi \right)=\frac{1}{T}|\int_{0}^{T}X_{t}e^{i\xi t}\;dt{|}^{2}\;.$$ (18)

The maximum amplitude of the PSD corresponds to the most prominent oscillation and it is given by

$$F\left({\left\{X_{t}\right\}}_{t=0}^{T}\right)=\max_{\xi }{\left|\hat{X}\right|}^{2}\left(\xi \right)\;.$$ (19)

This observable is not in the form of (Eq 1); however, our stationary sensitivity analysis as presented in (Eq 4)–(Eq 8) still applies; in order for the SB in (Eq 4) to be independent of the final time *T* in the stationary regime, we have to prove that the variance of *F* scales like $O(\frac{1}{T})$. Indeed,

$$\begin{array}{c} \text{Var}\left[F\left({\left\{X_{t}\right\}}_{t=0}^{T}\right)\right]=E\left[F{\left({\left\{X_{t}\right\}}_{t=0}^{T}\right)}^{2}\right]-E{\left[F\left({\left\{X_{t}\right\}}_{t=0}^{T}\right)\right]}^{2}\\ \leq \frac{1}{T^{2}}E\left[\overset{T}{\underset{0}{\int }}\left|X_{t}\right|\;dt\right], \end{array}$$ (20)

showing that the variance of *F* is of order $O(\frac{1}{T})$, provided $E|X_{t}|$ remains bounded.

**Fig 2:**
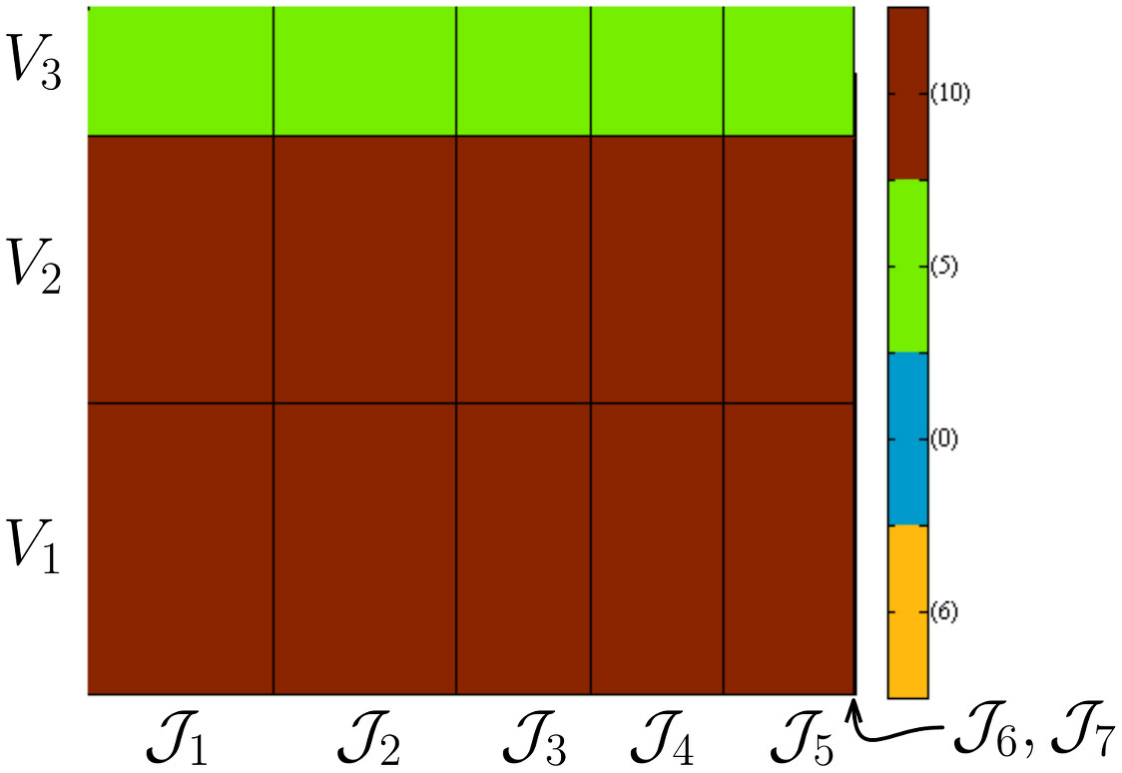
The SB for the SI *S*_*k*, ℓ_, see Eq (4), can be written as $|S_{k,\ell }|<V_{\ell }J_{k}$, where $V_{\ell }=\sqrt{\text{Var}(F_{\ell })}$ and $J_{k}=\sqrt{I_{kk}(Q_{\left[0,T\right]}^{\theta })}$. In this figure, the x-axis is divided into *K* = 7 intervals each with size $J_{k}$ and the y-axis into 3 intervals each having size *V*_ℓ_. The model is the p53 and the observables chosen here is the maximum amplitude of the PSD (Eq (19)) for the 3 species. The area of the (*k*, ℓ) rectangle corresponds to the SB of *S*_*k*, ℓ_. The red, green, blue and orange rectangles correspond to values of the SB larger than 5000, between 5000 and 500, between 500 and 50 and less than 50, containing 10, 5, 0, and 6 SIs, respectively. Notice that the 6-th and 7-th parameters are relatively insensitive since the corresponding pathwise FIM elements are relatively very small.

In total, 21 SIs which correspond to 3 observables (max amplitude of PSD for each species time-series) and 7 parameters must be computed. In Fig 2, the stationary SB (Eq (6)) of the SIs of the 3 observables with respect to the 7 parameters is plotted as rectangle; the x-axis is divided into *K* = 7 intervals each with size the square root of the pathwise FIM (Eq (8)) while the the y-axis is divided into *L* = 3 intervals each with size equal to the square root of the IAT (Eq (7)). The area of the rectangle corresponds to the arithmetic value of the SB. Thus, rectangles with small area indicate that the corresponding SI should also be small. In Fig 2, the two least sensitive parameters have relatively so small SBs, due to the fact that the FIM for the 6th and 7th parameter is relatively small, therefore they cannot be distinguished in the plot.

Notice that the grouping of the SIs of Fig 2 corresponds to the case “Setting a pre-specified tolerance” as described in the “How to use the strategy” section. Dictated by the average value of the observable functions which take values in the range between 10^3^ and 10^4^, we set the tolerance values to 5000, 500 and 50.

**Fig 3:**
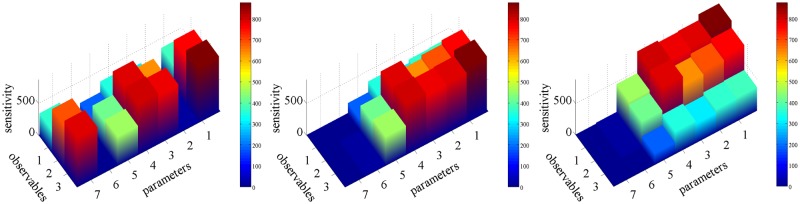
SIs for the maximum value of the PSD (Eq (19)) of the three species of the p53 model for *t* ∈ [0, 50] computed using the coupling gradient estimator (Eq (12)). No ordering of the observables or the parameters in the left plot. In the middle plot, the SIs are ordered in the parameter direction using the pathwise FIM (Eq (8)). The ordering reveals that the pathwise FIM can serve as a first screening procedure to exclude the insensitive parameters. In the right plot, the estimated sensitivities are further ordered in the observable direction by sorting the IAT (Eq (7)) in descending order.

In order to validate the ordering of the SIs from high to low values based on the stationary SB, we estimate them using the finite-difference coupling gradient estimator. In the left plot of Fig 3, the SIs are plotted without any ordering, i.e., as they are provided by the database [49]. In the middle plot, the SIs are ordered in the parameter direction according only to the values of pathwise FIM. As expected from Fig 2 and the SB, the two least sensitive parameters have relatively very small SIs. In the right plot of Fig 3, the SIs are further ordered according to the values of IAT. The SIs with the largest values are concentrated to the right upper corner which correspond to the larger SBs validating *Step 1* of the proposed strategy. Moreover, despite the fact that the SB correctly predicts the ordering of the SIs, the actual values of the SIs which correspond to the SB that are labeled sensitive (red color in Fig 2) differ by an order of magnitude from the values of the SB making the *Step 2* of the proposed strategy necessary for quantitative sensitivity results. On the other hand, the SIs that are labeled as insensitive (dark blue color in Fig 2) can be safely eliminated from the *Step 2* since the SB are relatively close to zero (when compared to the remaining value of SB).

### An EGFR model

The EGFR model is a well-studied reaction network describing signaling phenomena of (mammalian) cells [32–34]. As its name suggests, EGFR regulates cell growth, survival, proliferation and differentiation and plays a complex and crucial role in embryonic development and in tumor progression [50, 51]. In this paper, we study the reaction network developed by Kholodenko et al. [52] which consists of 23 species and 47 reactions.

The propensity function for the *R*_*j*_ reaction of the EGFR network is written in the form (mass action kinetics, see [7])

aj(x)=kj(xAjαj)(xBjβj),j=1,…,47andj≠7,14,29, (21)

for a reaction of the general form “$\alpha_{j}A_{j}+\beta_{j}B_{j}\overset{kj}{\to }\ldots$”, where *A*_*j*_ and *B*_*j*_ are the reactant species, *α*_*j*_ and *β*_*j*_ are the respective number of molecules needed for the reaction and *k*_*j*_ the reaction constant. The binomial coefficient is defined by (nk)=n!k!(n−k)!. Here, **x**_*A*_*j*__ and **x**_*B*_*j*__ is the total number of species *A*_*j*_ and *B*_*j*_, respectively. Reactions *R*_7_, *R*_14_, *R*_29_ are exceptions with their propensity functions being described by the Michaelis–Menten kinetics, see [7],

$$a_{j}\left(x\right)=V_{j}x_{A_{j}}/(K_{j}+x_{A_{j}}),\;j=7,14,29\;,$$ (22)

where *V*_*j*_ represents the maximum rate achieved by the system at maximum (saturating) substrate concentrations while *K*_*j*_ is the substrate concentration at which the reaction rate is half the maximum value. The parameter vector contains all the reaction constants,

$$\theta ={\left[k_{1},\ldots ,k_{6},k_{8},\ldots ,k_{13},k_{15},\ldots ,k_{28},k_{30},\ldots ,k_{47},V_{7},K_{7},V_{14},K_{14},V_{29},K_{29}\right]}^{T}\;,$$ (23)

with *K* = 50. In this study the values of the reaction constants are the same as in [52].

**Fig 4:**
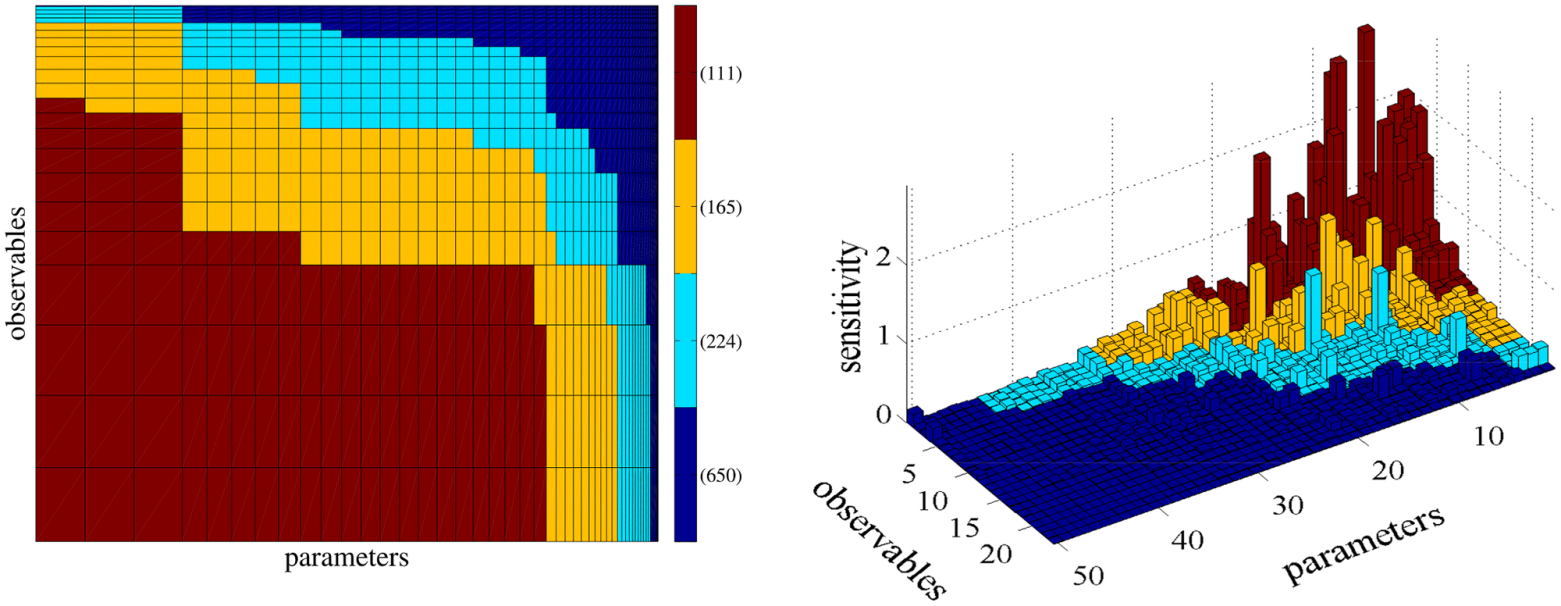
*Step 1* of the proposed sensitivity analysis strategy, graphically presented in the left plot, consists of identifying the least sensitive SIs (see also Fig 2). The area of rectangle (*k*, ℓ) represents the SB of the SI, *S*_*k*, ℓ_, for the transient regime. Every color corresponds to a predefined percentage of the total area, i.e. blue, light blue, yellow and red correspond to to 10%, 15%, 25% and 50% of the total area containing 650, 224, 165 and 111 SIs, respectively. The plot in the right represents *Step 2* of the strategy where each column corresponds the a SI which was estimated by the coupling estimator (Eq (12)). The computed SIs are colored by the region identifier color of the left plot, showing that areas with low sensitivities correspond to areas with low values of the SB.

Since the EGFR reaction network models signalling phenomena, it consists of a transient regime that corresponds to the time interval [0, 50] and a stationary regime which approximately corresponds to the time interval [50, ∞). In this study, the computations in the steady states regime were done in the time interval [50,100]. The general SB (Eq (4)) and the stationary SB (Eq (6)) are employed for the transient and the stationary regime, respectively. Fig 4 presents results from the transient regime. In the left plot of Fig 4, the parameters (x-axis) and species populations (y-axis), which define the observable functions, are sorted according to the square root of pathwise FIM and the square root of the variance of the observable, respectively, as dictated by the SB (see also Fig 2). Thus, the area of every rectangle corresponds to the value of the SB. This plot visualizes *Step 1* of the proposed strategy; the SI, *S*_*k*, *l*_, corresponding to a rectangle with small area can be safely excluded from *Step 2* of the sensitivity analysis strategy. The coloring of the rectangles is performed as follows: starting from rectangles with large area we color the first 50% of the total area using red, the next 25% using yellow and the next 15% and 10% using light blue and dark blue, respectively. This grouping is equivalent to “Setting the maximum number of SIs” as discussed in section “How to use the proposed strategy”.

In the right plot of Fig 4, *Step 2* of the proposed strategy is depicted. Even though all the SIs are computed using the coupling method for validation purposes, we could exclude for instance the SIs that correspond dark blue area in the left plot of Fig 4 since these “dark blue” SIs are just a small portion of the total area of the rectangle. Thus, approximately half of the SIs are excluded from *Step 2* and a upper bound (or tolerance) is assigned to them. Moreover, the right plot of Fig 4 serves as a validation of the proposed strategy; for instance, the SIs with large values are concentrated on the upper right corner (red and yellow in the right plot of Fig 4) which corresponds to the large values of the SB while the SIs with small values (dark blue) are concentrated on the lower left corner validating the proposed strategy.

**Fig 5:**
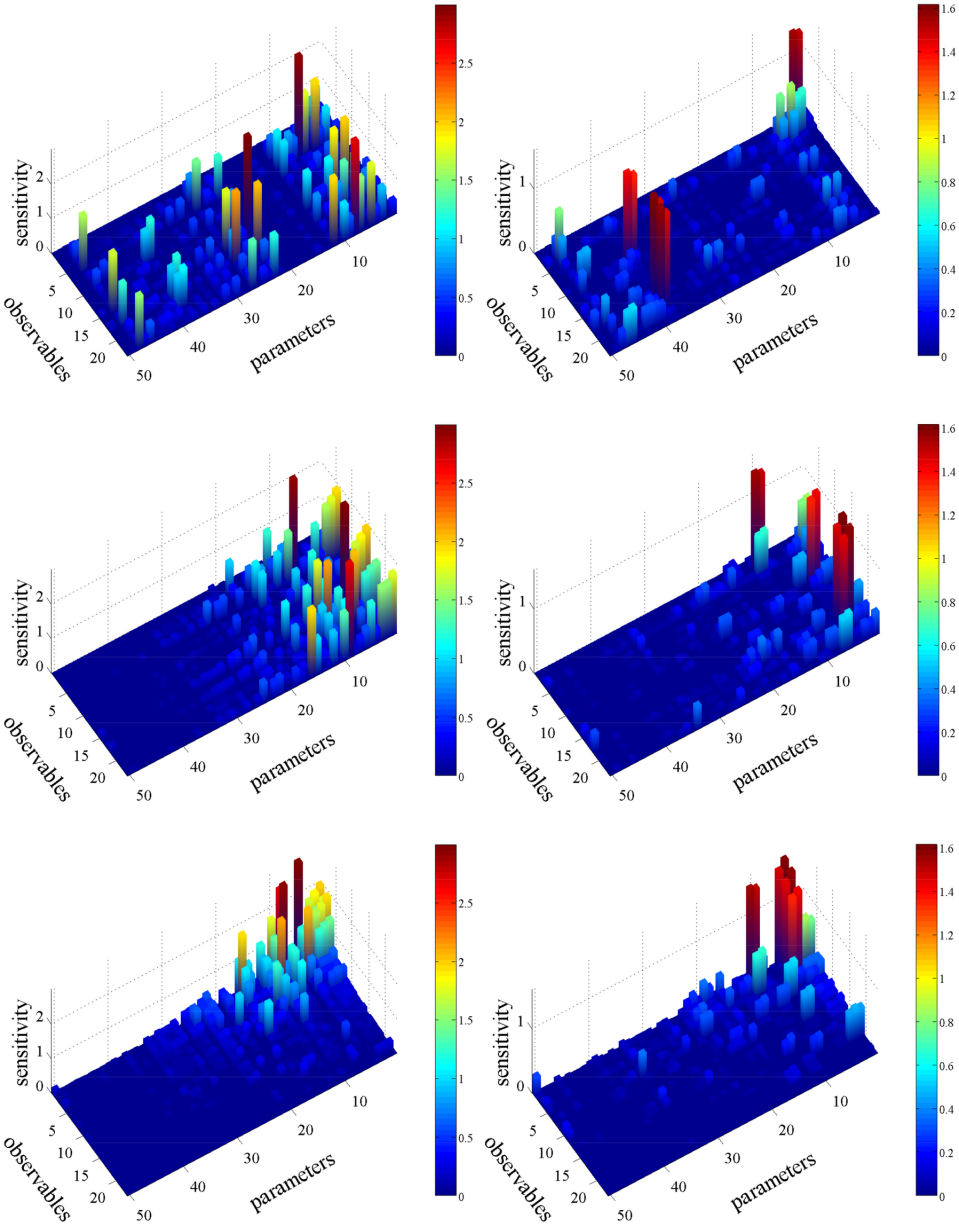
SIs for the EGFR model on the transient regime *t* ∈ [0, 50] (plots on left column) and on steady states regime *t* ∈ [50, 100] (plots on right column). The upper row presents the SIs without any ordering. On the middle row, the SIs are ordered in the parameter direction using the pathwise FIM (Eqs (10) and (8) for the transient and stationary regime, respectively). On the lower row, the SIs are further ordered using the standard deviation of the observable and the IAT for the transient and stationary regime, respectively.

In Fig 5, the SIs for the transient regime (plots on left column) and the stationary regime (plots on right column) are presented showing that the proposed strategy is capable of handling both regimes. For further validation, all SIs are computed using the finite-difference coupling estimator (Eq (12)). In the upper row of Fig 5, the SIs are unordered (arranged according to the database ordering, see [49]). In the middle row of Fig 5, the SIs are ordered in the parameter direction using only the pathwise FIM given by (Eq 10) for the transient regime and by (Eq 8) for the stationary regime. Notice that this ordering produces also a qualitative separation between insensitive and sensitive parameters. Hence, pathwise FIM alone can serve as an even simpler alternative to *Step 1* of the proposed strategy (see also the Discussion section, below). In the lower row of Fig 5, the SIs are further ordered using the standard deviation of the time-averaged observable and the IAT for the transient and stationary regime, respectively. In both regimes, the SIs with large values are concentrated on the upper right corner (lower row of Fig 5) which corresponds to the large values of the SB validating the proposed strategy. Finally, notice that there are SIs for insensitive parameters (left side in lower row’s plots) with relatively non-negligible values, however they stem from the statistical bias of the coupling method and not from a wrong labelling of the SIs based on the SB.

### A protein homeostasis model

In [35], the authors propose a reaction network that models the role of two chaperones, the Hsp70 and the Hsp90, in the maintenance of protein homeostasis. Loss of protein homeostasis is the common link between many neuro-degeneration disorders which are characterized by the accumulation of aggregated protein and neuronal cell death. The authors examined the role of both Hsp70 and Hsp90 under three different conditions: no stress, transient stress and high stress. Their model was validated against experimental data. The studied reaction network consists of 52 species and 80 reactions with propensities being of mass action kinetics type described by (Eq 21). The reaction constants as well as the initial populations were taken from [35]. Defining as observables the averaged species populations, i.e., $f_{\ell }(x)=x_{\ell },\;l=1,\ldots ,N$ in (Eq 1), the total number of SIs in this model is 4160. The parameter vector consists of all reaction constants

$$\theta ={\left[k_{1},\ldots ,k_{80}\right]}^{T}$$ (24)

where the parameter values used here were taken from [35].

**Fig 6:**
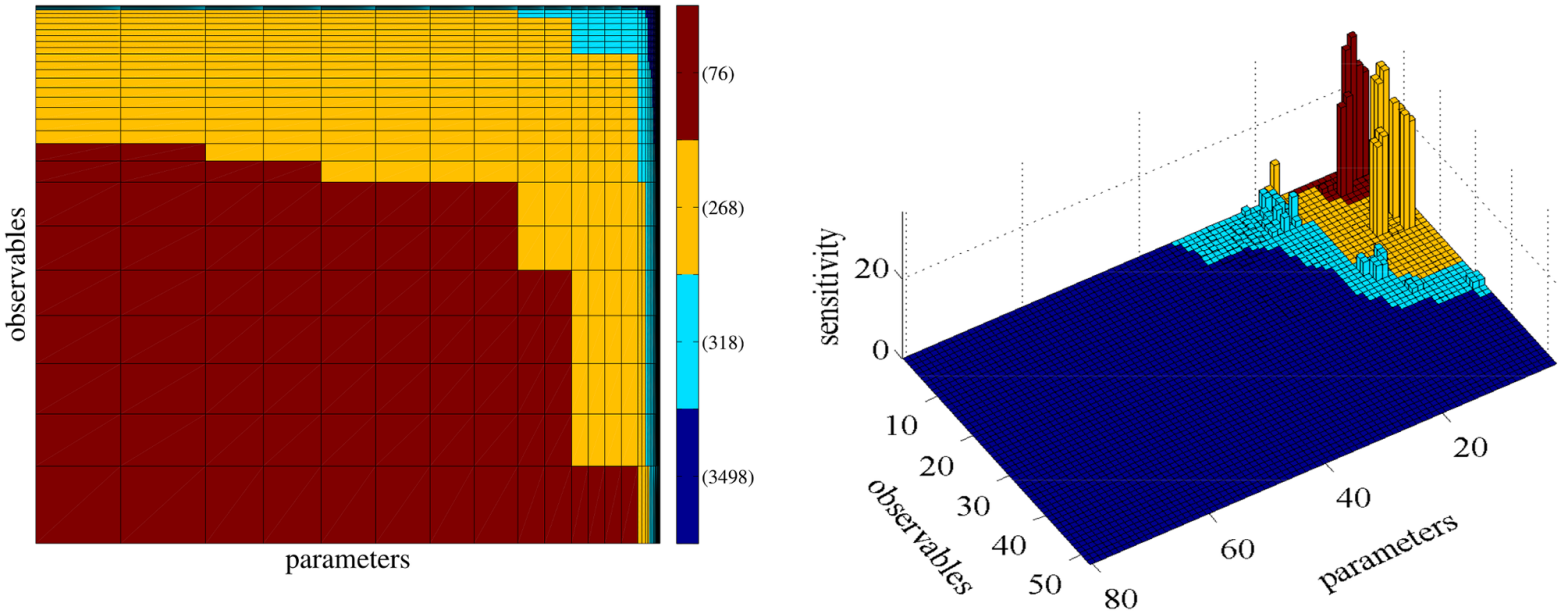
*Step 1* of the proposed sensitivity analysis strategy consists of identifying the least sensitive parameters. In the left plot, the area of rectangle (*k*, ℓ) is the SB of the SI, *S*_*k*, ℓ_ (see also Fig 2). Red, yellow, light blue and dark blue regions correspond to SB values greater than 100, between 10 and 100, between 1 and 10 and less than 1, containing 76,268,318 and 3498 SIs, respectively. The plot in the right shows *Step 2* of the strategy and simultaneously serves as a validation of the proposed methodology. Each column correspond to an actual SI, computed by the coupling method. The SI are sorted according to the sorting provided by the the first step of the strategy and colored by the region identifier color of the left plot, showing that areas with low sensitivities correspond to areas with low values of the SB.

In this model under the mechanism of no stress, only few SIs have large values while most of them are close to zero presenting a good example for “sloppiness” (see right plot of Fig 6). Note that when more complex mechanisms are included, the “sloppiness” of the reaction network will be changed but since we are interested in the validation of the proposed strategy, we restrict our discussion in the no stress case. Due to the high number of SIs, it is of great importance to screen out the insensitive pairs of parameters–observables using the stationary SB (Eq (6)). Then, a more accurate and refined estimation of the potentially large SIs using the coupling method can be performed (*Step 2* of the proposed strategy).

We group the SIs in the same way as in the p53 model, i.e. the range of the estimated SB is divided into regions of the same order of magnitude, e.g. the red and the dark blue region on the left plot of Fig 6 correspond to SIs in which the SB has a value greater than 100 and less that 1, respectively. The SIs with large values are concentrated on the upper right corner (right plot of Fig 6) which corresponds to the large values of the SB while the SIs with small values are concentrated on the lower left corner, validating once again the proposed strategy. More precisely, *Step 1* of the proposed sensitivity strategy consists of screening out the parameter–observable pairs that correspond to regions of rectangles with small area. Assigning the value of the SB to the SIs with associated SB value less than 1 (dark blue in Fig 6) results in a significant reduction in the total computational cost since 3498 out of 4160 (approximately 85%) SIs can be safely discarded as insensitive. Then, in *Step 2*, the SIs of the remaining pairs are computed using the coupling method. As it can be seen in the right plot of Fig 6, computing the SIs in red, yellow and light blue areas is enough to obtain the important information for the whole sensitivity matrix.

## Discussion

This section provides a detailed discussion on the computational gain of the proposed strategy as well as a simple formula on the achieved speedup. Moreover, we discuss the error quantification in the proposed sensitivity analysis strategy.

### Using (only) FIM to screen out insensitive parameters

As discussed earlier (middle row of Fig 5) the pathwise FIM (Eqs (10) or (8) in the steady state case) can serve as a fast alternative screening method instead of the complete SB (inequalities (4) or (6) in the steady state case). In this case, the calculation of the standard deviation of the observable (or the IAT (Eq (7)) in the stationary case) is bypassed leading to a less accurate but observable-independent screening method. The ordering of the FIM from high to low values provides a qualitative measure of sensitivity with respect to the parameters.

**Fig 7:**
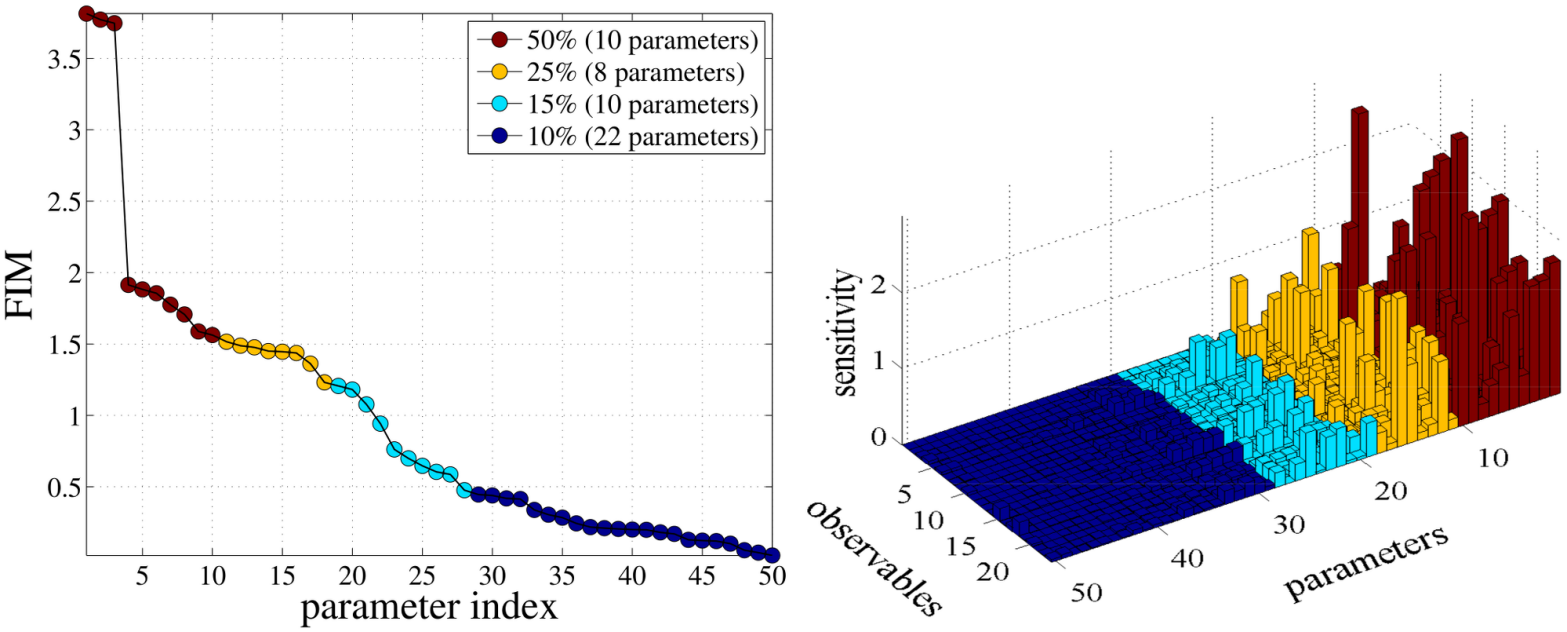
An alternative 1st step in the proposed strategy is presented in left plot where the diagonal elements of the pathwise FIM for the EGFR model is sorted in descending order. This ordering of the pathwise FIM gives a sorting in the parameters which gives a qualitative measure of the sensitivity of the model with respect to parameters. In the second step of the strategy (right plot), the SIs computed using the finite-difference coupling estimator are ordered according to the pathwise FIM value of the left plot. This figure validates the fact that pathwise FIM alone can give a first qualitative estimate of the SIs.

In Fig 7, the pathwise FIM alone is utilized for the ordering of the SIs for the EGFR model in the stationary regime, providing a computationally less expensive alternative to *Step 1* of the strategy discussed earlier. In the left plot of Fig 7, the pathwise FIM is ordered in descending order. Then, the potentially most sensitive parameters whose pathwise FIM values summing to 50% of the total sum of the pathwise FIM are organized into the red group and the following 25%,15% and 10% are organized into three groups (yellow, light blue and dark blue, respectively). Notice that this grouping is not unique and different parameter groupings can be used depending on the model under consideration. In the right plot of Fig 7, the SIs are computed using the coupling method and sorted according to the ordering given by the pathwise FIM as in the middle row of Fig 5. Moreover, the SIs are grouped and colored according to the grouping based on the pathwise FIM, see left plot of Fig 7. It is evident that there is a separation of the SIs into groups containing parameters with high (red and yellow), low (light blue) and almost zero (dark blue) sensitivities.

Although pathwise FIM as a sensitivity tool for reaction networks was studied and used earlier in [25], there are some differences with the methodology proposed in this article. Here pathwise FIM serves as part of the upper bound of the SI while in [25] there was no (immediate) connection of the pathwise FIM with the SIs. Moreover, in [25] there was no estimation of the actual SIs while here only the SIs of the most sensitive species/parameters are estimated in *Step 2* of the proposed strategy. Nevertheless, estimates such as (Eq 4) and (Eq 26) below give us quantified guarantees to employ only the pathwise FIM, thus bypassing the costly *Step 2*. Of course, we can only use this strategy provided that identifying and screening out insensitive parameters is the focus of the sensitivity analysis. We also refer to the “Computational Cost” subsection below for related comments on this issue.

### Error quantification in the accelerated sensitivity analysis

In this section, we quantify the error of the proposed methodology under the assumptions that only pathwise FIM is used in *Step 1* and the quantity of interest is *f*_ℓ_(**x**) = *x*_ℓ_, ℓ = 1, …, *N*, where *N* is the number of the species. We consider the case where there is a fixed amount of computational resources *M* = *K*′ × *N*, where *K*′ is the maximum number of parameters in which SIs can be computed. Note that this is a special case of the error quantification discussed in section “How to use the proposed strategy”. Then the questions posed here are:

(a) For which parameters should the SIs be computed?
(b) What is the magnitude of the SIs that are discarded by *Step 1* of the proposed strategy?

To answer these questions we define the set

$$C_{K^{\prime }}:=\{\text{all}\;k:k\text{-th}\;\text{diagonal}\;\text{element}\;\text{of}\;\text{pathwise}\;\text{FIM}\;\text{is}\;\text{one}\;\text{of}\;\text{the}\;K^{\prime }\;\text{largest}\;\text{values}\}$$ (25)

Then, the answer to (a) is to compute SIs for all *k* ∈ *C*_*K*′_. The answer to (b) is that for all *k* ∉ *C*_*K*′_ and ℓ = 1, …, *N*, we have the bound

$$|S_{k,\ell }|\leq \sqrt{\tau_{\mu^{\theta }}\left(f_{\ell }\right)}\max_{k\notin C_{K^{\prime }}}\sqrt{I_{H}{\left(Q^{\theta }\right)}_{k,k}}:=\mathrm{TOL}\left(\ell ,K^{\prime }\right)\;,$$ (26)

where the tolerance $\text{TOL}(\ell ,K^{\prime })$ is defined as above and the last inequality follows from the stationary SB (ineq. (6)). The SB given by (Eq 26) assures that the error in the proposed methodology due to the discarded (by *Step 1*) SIs, will be less or equal to ${\text{max}}_{\ell }\text{TOL}(\ell ,K^{\prime })$.

### Computational Cost

The computational cost of the proposed strategy, consists of the cost of the estimation of the SB (ineq. (4) or (6)) as well as the cost of estimation of the SIs not discarded from *Step 1* by using the coupling method. This cost is compared with the computational cost of using the coupling method for all SIs without the screening step of *Step 1*. The comparison will be done in terms of the computational gain *G*, which is the sum of the costs from *Step 1* and *Step 2* of the strategy over the cost of the coupling method for all SIs:

$$\begin{array}{l} G:=\frac{\text{cost}\;\text{of}\;\text{proposed}\;\text{strategy}}{\text{cost}\;\text{of}\;\text{computing}\;\text{all}\;\text{SIs}}\\ \;\;\;=\frac{\text{cost}\;\text{of}\;\text{SB}}{\text{cost}\;\text{of}\;\text{computing}\;\text{all}\;\text{SIs}}+\frac{\text{cost}\;\text{of}\;\text{computing}\;\text{sensitive}\;\text{SIs}}{\text{cost}\;\text{of}\;\text{computing}\;\text{all}\;\text{SIs}}\\ \;\;\;=G_{1}+G_{2}\;. \end{array}$$ (27)

Note that 1/G measures how many times the proposed strategy is faster compared to the estimation of all SIs using the coupling method. For the technical details on this comparison see S4 File.

For simplicity we consider the case where only the pathwise FIM is used to discard insensitive parameters, see the previous section “Error quantification in the accelerated sensitivity analysis”. In this case information on the sensitivity of observables is obtained from (Eq 26) using *Step 1*. Moreover, the quantity of interest (observables) considered here is the species population, i.e. *f*_ℓ_(**x**) = *x*_ℓ_. Finally, the comparison is being done under the requirement that the *relative confidence intervals* (for the definition, see S4 File) of all estimators involved in both approaches will have variance less or equal to *δ* ≪ 1.

We observe that the variance of the SB is much less than the variance of the coupling method (i.e. Eq (13)) leading to *G*_1_ ≪ 1 (see Figure A in S4 File). After the computation of the SB, the SIs are grouped into two categories, i.e., potentially sensitive and insensitive. Let *K*′ be the number of sensitive parameters that correspond to the potentially sensitive SIs and *K* the number of all parameters. Then, under some reasonable assumptions (see S4 File for more details), the *G*_2_ term is approximately equal to $G_{2}=\frac{K^{\prime }}{K}$ and the computational gain *G* is approximated by

$$G\approx \frac{K^{\prime }}{K}.$$ (28)

We next validate the approximation (Eq 28) of the computational gain on the EGFR model presented earlier, where the number of parameters is *K* = 50. By inspection of Fig 7 the number of sensitive parameters is *K*′ = 28, which correspond to the first three colored regions of the graph. Thus, *G* can be approximated by $G\approx \frac{28}{50}=0.56$. On the other hand, we compute the *G*_1_ and *G*_2_ terms exactly by measuring the simulation cost of the two methods in terms of counting the number of required samples. The *G*_1_ term is equal to 0.0034 showing that the estimation of the SB needs about 300 times less samples than that of computation of SIs using the coupling estimator while *G*_2_ is equal to 0.72. As a result, the actual speed-up due to the proposed strategy is approximately 1/G ≈ 1.4. These are modest computational gains, however they are achieved with minimal investment in computational resources in *Step 1*; on one hand, the variance calculation in the SB is anyhow necessary in any forward simulation, in order to obtain confidence intervals for the species (observables), while the calculation of the pathwise FIM is straightforward and can be viewed as the simulation of just one additional observable.

**Fig 8:**
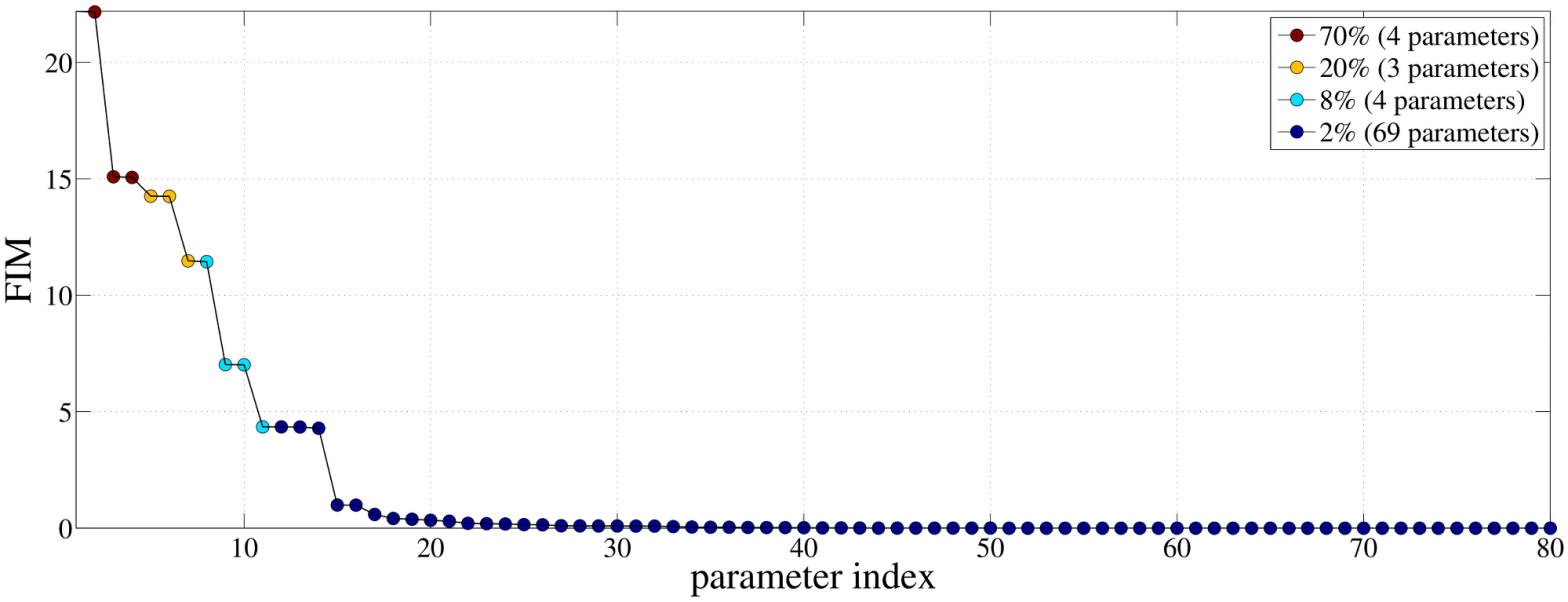
Diagonal elements of the pathwise FIM for the Protein Homeostasis model sorted in descending order. The coloring of the parameters is being done as follows: parameters whose pathwise FIM values sum to 70%, 20%, 8% and 2% of the total sum of pathwise FIM values, are grouped together and correspond to red, yellow, light blue and dark blue color, respectively.

Although the computational gains in systems such as EGFR, where a large number of parameters are relatively sensitive are modest, the gains are very significant in “sloppy” systems. Indeed, for the Protein Homeostasis reaction network, assuming that *G*_1_ is negligible compared to *G*_2_ and using the approximation in (Eq 28) to obtain an estimate for *G*_2_, we have that the computational gain for this model is $G\approx \frac{K^{\prime }}{K}=\frac{16}{80}=0.2$. The value for *K*′ is obtained by assuming that the important parameters are those colored with red, yellow and light blue in Fig 8. The value of *G* ≈ 0.2 suggests a 5-times speed-up in the sensitivity analysis of this “sloppy” example.

Finally, as discussed at the end of the subsection “Using (only) FIM to screen out insensitive parameters”, we may also employ just *Step 1* (and skip *Step 2*), at least when identifying and screening out insensitive parameters is the focus of the sensitivity analysis. This may be the case in high-dimensional reaction networks with suspected “sloppy” characteristics. In this case, the computational cost of the proposed methodology dramatically decreases. For instance, in the EFGR case the speed-up is 1/*G*_1_ ≈ 294 times faster than the full coupling method. In the case of the Protein Homeostasis example the gains are much higher.

## Conclusions

Existing information-based parametric sensitivity analysis methods for stochastic reaction networks can tackle systems with a large number of parameters without however providing insights on specific quantities of interest (observables). On the other hand, existing gradient-based methods can perform accurate sensitivity analysis for arbitrary observables but with high computational cost which can become prohibitive for networks with a high dimensional state and/or parameter space due to (a) the high variance of SI estimators, and/or (b) the need to calculate gradients corresponding to all parameters. In the proposed methodology, we address these challenges through a two-step strategy, combining two different sensitivity analysis approaches using a new SI upper bound. More specifically, in *Step 1*, we first perform an “insensitivity” analysis: namely, the parameters’ sensitivity can be systematically screened and many can be eliminated as insensitive based on derived SBs of the SI which are computationally inexpensive; in *Step 2*, only the potentially sensitive parameters which were not screened out in *Step 1* are estimated exactly, based on the finite-difference (gradient) coupling approach.

The acceleration in sensitivity analysis due to the proposed strategy can be very significant especially when sloppy systems are considered and most of the parameters are expected to be screened out as insensitive from *Step 1*. Moreover, the proposed strategy offers a simple way to rationally balance accuracy and computational cost, selecting the number of insensitive parameters that need to be discarded from further sensitivity analysis. Specifically, the tradeoff between computationally expensive gradient estimation and accuracy in SI computation is quantified in terms of an easily computable SBs on the SIs (see (Eq 4) and (Eq 6) for the transient and the stationary regimes, respectively), which in turn determines a cutoff (or a user-determined tolerance) of insensitivity. The proposed strategy, through the SB, guarantees that the SIs for the insensitive parameters will lie below the value of the cutoff. Thus, it is upon the practitioner’s choice how many of the parameters will be screened out, based on the SB values and the overall computational budget. The computational acceleration of the proposed strategy is approximately quantified by the ratio between the total number of parameters over the number of the potentially sensitive parameters which were not eliminated in *Step 1*, i.e., the ratio $\frac{K}{K^{\prime }}$.

We conclude this paper by noting that the proposed strategy is by no means restricted to well-mixed systems such as reaction networks and can be directly applied to spatially-extended systems (high-dimensional in state space). Indeed, the pathwise FIM used in the screening in *Step 1*, still has low variance for spatially-extended systems such as Kinetic Monte Carlo, as it has been shown in catalysis examples [24], while the coupling method in *Step 2* can be modified for such models so that it still gives reduced-variance estimators for the SIs, see [14]. In fact, the proposed strategy is absolutely necessary in spatially-expended systems with a large number of parameters since gradient computations are prohibitively expensive due to the need for a large number of repeated runs arising in the computation of SIs. Finally, the proposed strategy is compatible with any other sensitivity analysis approach in the sense that any gradient estimation method can be utilized in *Step 2* instead of the coupling method.

## Supporting Information

S1 File Information Theory and Sensitivity Bounds. The SBs are presented from an information theory perspective. The general SBs (both transient and stationary) are obtained by a limiting process on the relative entropy between path distributions. The information theory perspective provides also intuitive and explicit formulas for the quantities of interest for both discrete-time Markov chains and continuous-time Markov chains.
(PDF)

S2 File Unbiased Statistical Estimators for pathwise FIM and IAT. (PDF)

S3 File Coupling of Stochastic Processes. A brief revision of the coupling method proposed by Anderson for the estimation of gradients in well-mixed reaction network systems.
(PDF)

S4 File Computation Cost based on Variance Estimates. An approximation of the variance of the SB is first presented. Then, we present the technical details for the computational cost comparison of the proposed strategy with the coupling method applied to all the SIs of the model.
(PDF)

S5 File Matlab code. A zip file that contains all the Matlab source files needed for the generation of the figures of this publication.
(ZIP)
