# Supplementary material for: Accelerated Sensitivity Analysis in High-Dimensional Stochastic Reaction Networks
Source: PLoS One. 2015 Jul 10;10(7):e0130825. doi: 10.1371/journal.pone.0130825 (PMC4498611; doi:10.1371/journal.pone.0130825)
Supplement: S1 File — The SBs are presented from an information theory perspective. The general SBs (both transient and stationary) are obtained by a limiting process on the relative entropy between path distributions. The information theory perspective provides also intuitive and explicit formulas for the quantities of interest for both discrete-time Markov chains and continuous-time Markov chains. (PDF) [file pone.0130825.s001.pdf]

Information Theory and Sensitivity Bounds

This supporting information file presents the sensitivity bounds (SBs) from an information theory perspective. The general SBs (both transient and stationary) are obtained by a limiting process on the relative entropy between path distributions. The information theory perspective provides also intuitive and explicit formulas for the quantities of interest for both Discrete Time Markov Chains (DTMC) and Continuous Time Markov Chains (CTMC).

1 Pathwise information theory and sensitivity bounds

Let the path space distribution of a stochastic process on the time-interval $[0,T]$ be denoted by $Q^{\theta}_{[0,T]}$ where $\theta \in \mathbb{R}^K$ is the parameter vector. The pathwise relative entropy of $Q^{\theta}_{[0,T]}$ with respect to $Q^{\theta+\epsilon}_{[0,T]}$ where $\epsilon \in \mathbb{R}^K$ corresponds to the perturbation vector is defined as

$$\mathcal{R}\left(Q^{\theta}_{[0,T]}\,|\,Q^{\theta+\epsilon}_{[0,T]}\right) = \mathbb{E}_{Q^{\theta}_{[0,T]}}\Big[\log\left(\frac{dQ^{\theta}_{[0,T]}}{dQ^{\theta+\epsilon}_{[0,T]}}\right)\Big] = \int \log\left(\frac{dQ^{\theta}_{[0,T]}}{dQ^{\theta+\epsilon}_{[0,T]}}\right) dQ^{\theta}_{[0,T]}\,, \tag{1}$$

where $\frac{dQ^{\theta}_{[0,T]}}{dQ^{\theta+\epsilon}_{[0,T]}}$ is the Radon-Nikodym derivative of unperturbed path distribution, $Q^{\theta}_{[0,T]}$, with respect to the perturbed path distribution, $Q^{\theta+\epsilon}_{[0,T]}$, while the integration is performed with respect to the probability measure, $Q^{\theta}_{[0,T]}$, [1]. A necessary condition for the relative entropy to be well-defined and finite is that the Radon-Nikodym derivative exists which is satisfied when $Q^{\theta}_{[0,T]}$ is absolutely continuous with respect to $Q^{\theta+\epsilon}_{[0,T]}$. Based on Girsanov formula , explicit formulas for the Radon-Nikodym derivative are provided in the following sections for both DTMC and CTMC. Even though not a distance but a divergence, relative entropy is always non-negative and it equals to zero if and only if the two distributions are equal almost everywhere. From an information theory perspective, relative entropy quantifies the loss of information when $Q^{\theta+\epsilon}_{[0,T]}$ is utilized instead of $Q^{\theta}_{[0,T]}$, [1].

Assume that the pathwise relative entropy, $\mathcal{R}\left(Q^{\theta}_{[0,T]}\,|\,Q^{\theta+\epsilon}_{[0,T]}\right)$, is smooth with respect to the parameter vector, θ. Then, in combination with its non-negativity property, pathwise relative entropy can be Taylor expanded around θ as

$$\mathcal{R}\left(Q^{\theta}_{[0,T]}\,|\,Q^{\theta+\epsilon}_{[0,T]}\right) = \frac{1}{2}\epsilon^T \mathcal{I}(Q^{\theta}_{[0,T]})\epsilon + O(|\epsilon|^3)\,, \tag{2}$$

where $\mathcal{I}(Q^{\theta}_{[0,T]})$ is the Hessian of the pathwise relative entropy which geometrically corresponds to the curvature around the minimum value. In the language of information theory, the $K \times K$ matrix, $\mathcal{I}(Q^{\theta}_{[0,T]})$, is the pathwise Fisher information matrix (FIM). An appealing property of the (pathwise) FIM is that it is independent of the perturbation vector, ϵ, nevertheless, it contains up to third order accuracy the sensitivity information as it is quantified by the (pathwise) relative entropy.

The sensitivity bound

$$|S_{k,l}| \leq \sqrt{\mathrm{Var}_{Q^{\theta}_{[0,T]}}(F_l)}\sqrt{\mathcal{I}(Q^{\theta}_{[0,T]})_{k,k}}\,, \tag{3}$$

is obtained by rearranging the generalized Cramer-Rao bound for a biased estimator, [2,3]. In particular, when the distribution in the Cramer-Rao theorem is the path distribution, the absolute value of the SI of the l-th observable is bounded by the inequality (3). An alternative and much more general proof of (3) has been recently shown in [4]. Indeed, for fixed final time, T, and $|\epsilon| \ll 1$ it was shown that for any bounded observable function, $F(\cdot)$,

$$\left|\mathbb{E}_{Q^{\theta+\epsilon}_{[0,T]}}[F] - \mathbb{E}_{Q^{\theta}_{[0,T]}}[F]\right| \le \sqrt{\mathrm{Var}_{Q^{\theta}_{[0,T]}}(F)}\sqrt{2\mathcal{R}\left(Q^{\theta}_{[0,T]}\,|\,Q^{\theta+\epsilon}_{[0,T]}\right) + O\left(\mathcal{R}\left(Q^{\theta}_{[0,T]}\,|\,Q^{\theta+\epsilon}_{[0,T]}\right)\right)} \;. \tag{4}$$

Letting $\epsilon = \epsilon_0 e_k, k = 1, \ldots, K$ where e_k are the k-th unit vector, dividing both sides with ϵ_0 and taking the infinitesimal perturbation limit $\epsilon_0 \to 0$, it holds for the sensitivity index (SI) of the l-th observable the SB inequality (3).

1.1 Stationary regime and sensitivity bounds

The SB at the stationary regime can be simplified and becomes independent of time for observables that are time-averaged quantities. Indeed, the pathwise relative entropy (consequently, the pathwise FIM) at the stationary regime is generically decomposed into a linear in time term plus a constant as follows

$$\mathcal{R}\left(Q^{\theta}_{[0,T]}\,|\,Q^{\theta+\epsilon}_{[0,T]}\right) = \mathcal{R}\left(\mu^{\theta}\,|\,\mu^{\theta+\epsilon}\right) + T\mathcal{H}\left(Q^{\theta}\,|\,Q^{\theta+\epsilon}\right) \;, \tag{5}$$

where $\mathcal{R}\left(\mu^{\theta}\,|\,\mu^{\theta+\epsilon}\right)$ is the relative entropy of the unperturbed stationary distribution, μ^{θ}, with respect to the perturbed, $\mu^{\theta+\epsilon}$, while $\mathcal{H}\left(Q^{\theta}\,|\,Q^{\theta+\epsilon}\right)$ is the Relative Entropy Rate (RER) which is independent of time, see [5,6] for more details.

On the other hand, as a consequence of the central limit theorem, the variance of the time-average observable $F(\{\mathbf{X}_t\}_0^T) = \frac{1}{T}\int_{t=0}^T f(\mathbf{X}_t)dt$ is of order $O(\frac{1}{T})$, hence, we define the quantity $\tau^T_{\mu^\theta}(f) := \frac{1}{T}\mathrm{Var}_{\mu^\theta}(TF)$ which scales $O(1)$ with respect to T. The limit

$$\tau_{\mu^\theta}(f) = \lim_{T\to\infty} \tau^T_{\mu^\theta}(f) \,,$$

is the Integrated Autocorrelation Time (IAT), [7]. Overall, the SB in (3) is rewritten at the stationary regime as

$$|S_{k,l}| \le \sqrt{\frac{1}{T}\mathrm{Var}_{Q^{\theta}_{[0,T]}}(TF_l)}\sqrt{\frac{1}{T}\left(T\mathcal{I}_{\mathcal{H}}(Q^\theta)_{k,k} + \mathcal{I}(\mu^\theta)_{k,k}\right)} \,,$$

where $\mathcal{I}(\mu^\theta)$ is the standard FIM of the steady state distribution μ^θ, [2]. Note that the pathwise FIM, $\mathcal{I}_{\mathcal{H}}(Q^\theta)$, is the Hessian of the RER, $\mathcal{H}\left(Q^{\theta}\,|\,Q^{\theta+\epsilon}\right)$. The pathwise FIM, $\mathcal{I}_{\mathcal{H}}(Q^\theta)$, has an analytical formula that can be calculated numerically; we refer to the next Sections–see also [5,6]–for more details. Consequently, sending $T \to \infty$, we obtain the stationary SB

$$|S_{k,l}| \le \sqrt{\tau_{\mu^\theta}(f)}\sqrt{\mathcal{I}_{\mathcal{H}}(Q^\theta)_{k,k}} \;. \tag{6}$$

Next, in Sections 2 and 3 we provide explicit and computationally tractable formulas for both IAT and pathwise FIM.

2 Discrete-time Markov chains

This section presents explicit formulas of the information theory quantities for the simpler case of DTMC. The analysis of the DTMC case serves (a) as a more intuitive and manageable example of stochastic processes and (b) as a intermediate step to handle the CTMC case. Next, let $\{\mathbf{x}_i\}_{i\in\mathbb{Z}^+}$ be a

discrete-time time-homogeneous Markov chain with separable state space E. The transition probability kernel of the Markov chain denoted by $P^\theta(\mathbf{x}, d\mathbf{x}')$ depends on the parameter vector $\theta \in \mathbb{R}^K$. Assume that the transition kernel is absolutely continuous with respect to the Lebesgue measure and the transition probability density function $p^\theta(\mathbf{x}, \mathbf{x}')$ is always positive for all $\mathbf{x}, \mathbf{x}' \in E$ and for all $\theta \in \mathbb{R}^K$. Exploiting the Markov property, the path space probability density $Q^\theta_{0,T}$ for the path $\{\mathbf{x}_i\}_{i=0}^T$ at the time horizon $0, 1, \ldots, T$ starting from the initial distribution $\nu^\theta(\mathbf{x}_0)$ is given by

$$Q^\theta_{0,T}(\mathbf{x}_0, \ldots, \mathbf{x}_T) = \nu^\theta(\mathbf{x}_0) p^\theta(\mathbf{x}_0, \mathbf{x}_1) \ldots p^\theta(\mathbf{x}_{T-1}, \mathbf{x}_T) \, .$$

We consider the perturbation by $\epsilon \in \mathbb{R}^K$ and the Markov chain $\{\tilde{\mathbf{x}}_i\}_{i\in\mathbb{Z}^+}$ with transition probability density function, $p^{\theta+\epsilon}(\mathbf{x}, \mathbf{x}')$, initial density, $\nu^{\theta+\epsilon}(\mathbf{x})$, as well as path distribution $Q^{\theta+\epsilon}_{0,T}$. Then, the Radon-Nikodym derivative of the unperturbed path distribution with respect to the perturbed path distribution takes the form

$$\frac{dQ^\theta_{0,T}}{dQ^{\theta+\epsilon}_{0,T}}(\{\mathbf{x}_i\}_{i=0}^T) = \frac{\nu^\theta(\mathbf{x}_0) \prod_{i=0}^{T-1} p^\theta(\mathbf{x}_i, \mathbf{x}_{i+1})}{\nu^{\theta+\epsilon}(\mathbf{x}_0) \prod_{i=0}^{T-1} p^{\theta+\epsilon}(\mathbf{x}_i, \mathbf{x}_{i+1})} \, ,$$

which is well-defined since the transition probabilities are always positive. The product representation of the path distributions results in an additive representation of the relative entropy of the path distribution, $Q^\theta_{0,T}$, with respect to the perturbed path distribution, $Q^{\theta+\epsilon}_{0,T}$. Indeed,

$$\begin{aligned}
\mathcal{R}\left(Q^\theta_{0,T} \,|\, Q^{\theta+\epsilon}_{0,T}\right) &= \int_E \cdots \int_E \nu^\theta(\mathbf{x}_0) \prod_{i=1}^T p^\theta(\mathbf{x}_{i-1}, \mathbf{x}_i) \log \frac{\nu^\theta(\mathbf{x}_0) \prod_{i=1}^T p^\theta(\mathbf{x}_{i-1}, \mathbf{x}_i)}{\nu^{\theta+\epsilon}(\mathbf{x}_0) \prod_{i=1}^T p^{\theta+\epsilon}(\mathbf{x}_{i-1}, \mathbf{x}_i)} d\mathbf{x}_0 \ldots d\mathbf{x}_T \\
&= \int_E \cdots \int_E \nu^\theta(\mathbf{x}_0) \prod_{i=1}^T p^\theta(\mathbf{x}_{i-1}, \mathbf{x}_i) \log \frac{\nu^\theta(\mathbf{x}_0)}{\nu^{\theta+\epsilon}(\mathbf{x}_0)} d\mathbf{x}_0 \ldots d\mathbf{x}_T \\
&+ \sum_{i=1}^T \int_E \cdots \int_E \nu^\theta(\mathbf{x}_0) \prod_{i=1}^T p^\theta(\mathbf{x}_{i-1}, \mathbf{x}_i) \log \frac{p^\theta(\mathbf{x}_{i-1}, \mathbf{x}_i)}{p^{\theta+\epsilon}(\mathbf{x}_{i-1}, \mathbf{x}_i)} d\mathbf{x}_0 \ldots d\mathbf{x}_T \\
&= \int_E \nu^\theta(\mathbf{x}_0) \log \frac{\nu^\theta(\mathbf{x}_0)}{\nu^{\theta+\epsilon}(\mathbf{x}_0)} d\mathbf{x}_0 + \sum_{i=1}^T \int_E \nu^\theta(\mathbf{x}_0) \prod_{i=1}^T p^\theta(\mathbf{x}_{i-1}, \mathbf{x}_i) \log \frac{p^\theta(\mathbf{x}_{i-1}, \mathbf{x}_i)}{p^{\theta+\epsilon}(\mathbf{x}_{i-1}, \mathbf{x}_i)} \mathbf{x}_0 \ldots d\mathbf{x}_i \\
&= \mathcal{R}\left(\nu^\theta \,|\, \nu^{\theta+\epsilon}\right) + \sum_{i=1}^T \mathcal{H}\left(\nu^\theta_i \otimes p^\theta \,|\, \nu^\theta_i \otimes p^{\theta+\epsilon}\right) \, ,
\end{aligned} \tag{7}$$

where $\mathcal{R}\left(\nu^\theta \,|\, \nu^{\theta+\epsilon}\right) := \mathbb{E}_{\nu^\theta}\left[\log \frac{\nu^\theta(\mathbf{x})}{\nu^{\theta+\epsilon}(\mathbf{x})}\right]$ is the relative entropy of the unperturbed initial distribution with respect to the perturbed one, while the quantity

$$\mathcal{H}\left(\nu^\theta_i \otimes p^\theta \,|\, \nu^\theta_i \otimes p^{\theta+\epsilon}\right) := \mathbb{E}_{\nu^\theta_i}\Big[\int_E p^\theta(\mathbf{x}, \mathbf{x}') \log \frac{p^\theta(\mathbf{x}, \mathbf{x}')}{p^{\theta+\epsilon}(\mathbf{x}, \mathbf{x}')} d\mathbf{x}'\Big] \, , \tag{8}$$

can be considered as the instantaneous relative entropy of the time-varying pathwise relative entropy. Moreover, we denote by the operator '$\otimes$' the product of two distributions (i.e., $\nu \otimes p(A \times B) := \int_A p(x, B)\nu(x)dx$). We also define

$$\nu^\theta_i(\mathbf{x}) := \int_E \cdots \int_E \nu^\theta(\mathbf{x}_0) p^\theta(\mathbf{x}_0, \mathbf{x}_1) \ldots p^\theta(\mathbf{x}_{i-1}, \mathbf{x}) dx_0 \ldots dx_{i-1} \, ,$$

which is the probability density function of the Markov chain at time instant i given that the initial distribution is ν^θ. The corresponding pathwise FIM, i.e., the Hessian of the pathwise relative entropy

(7), is given by

$$\mathcal{I}(Q^\theta_{0,T}) := \mathcal{I}(\nu^\theta) + \sum_{i=1}^{T} \mathcal{I}_\mathcal{H}(\nu_i^\theta \otimes p^\theta) \ , \tag{9}$$

where $\mathcal{I}(\nu^\theta) := \mathbb{E}_{\nu^\theta}[\nabla_\theta \log \nu^\theta(\mathbf{x}) \nabla_\theta \log \nu^\theta(\mathbf{x})^T]$ is the FIM of the initial distribution while

$$\mathcal{I}_\mathcal{H}(\nu_i^\theta \otimes p^\theta) := \mathbb{E}_{\nu_i^\theta}[\int_E p^\theta(\mathbf{x},\mathbf{x}') \nabla_\theta \log p^\theta(\mathbf{x},\mathbf{x}') \nabla_\theta \log p^\theta(\mathbf{x},\mathbf{x}')^T dx'] \ , \tag{10}$$

is the instantaneous FIM associated to the instantaneous relative entropy, $\mathcal{H}\left(\nu_i^\theta \otimes p^\theta \,|\, \nu_i^\theta \otimes p^{\theta+\epsilon}\right)$.

2.1 Stationary regime

In the stationary regime, the initial distribution is the stationary distribution, $\mu^\theta(\cdot)$, and the formula of the relative entropy is simplified to (5). The Relative Entropy Rate (RER), $\mathcal{R}\left(Q^\theta_{0,T} \,|\, Q^{\theta+\epsilon}_{0,T}\right)$, in the DTMC case is defined by, [5],

$$\mathcal{H}\left(Q^\theta \,|\, Q^{\theta+\epsilon}\right) = \mathbb{E}_{\mu^\theta}\Big[\int_E p^\theta(\mathbf{x},\mathbf{x}') \log \frac{p^\theta(\mathbf{x},\mathbf{x}')}{p^{\theta+\epsilon}(\mathbf{x},\mathbf{x}')} d\mathbf{x}'\Big] \ . \tag{11}$$

Then, it holds that $\mathcal{H}\left(\nu_i^\theta \otimes p^\theta \,|\, \nu_i^\theta \otimes p^{\theta+\epsilon}\right) = \mathcal{H}\left(\mu^\theta \otimes p^\theta \,|\, \mu^\theta \otimes p^{\theta+\epsilon}\right) = \mathcal{H}\left(Q^\theta \,|\, Q^{\theta+\epsilon}\right)$ for all $i = 1,...,T$. In other words, the instantaneous relative entropy is constant in the stationary regime and it is equal to the RER. Similar considerations hold for the FIM associated to the RER whose explicit formula is given by, [5],

$$\mathcal{I}_\mathcal{H}(Q^\theta) = \mathbb{E}_{\mu^\theta}\left[\int_E p^\theta(\mathbf{x},\mathbf{x}) \nabla_\theta \log p^\theta(\mathbf{x},\mathbf{x}') \nabla_\theta \log p^\theta(\mathbf{x},\mathbf{x}')^T d\,\mathbf{x}'\right] \ . \tag{12}$$

2.2 Integrated autocorrelation time for DTMC

Regarding the definition of the IAT, the difference between the continuous time (see below) and the discrete time cases is that the integral is replaced by a sum, i.e., for an observable function, f, IAT is given by, [7],

$$\tau_{\mu^\theta}(f) = \sum_{i=-\infty}^{\infty} < f(\mathbf{x}_i) - \mathbb{E}_{\mu^\theta}[f], f(\mathbf{x}_0) - \mathbb{E}_{\mu^\theta}[f] >_{\mu^\theta} \ . \tag{13}$$

3 Continuous-time Markov chains

Let $N_t = (N_t^1, \ldots, N_t^J)$ be a J-variate point process where N_t^j are independent Poisson processes with predictable stochastic intensity $\lambda_t^j = a_j^\theta(X_{t-})$, see [8]. The Radon-Nikodym derivative of the path distribution, $Q^\theta_{[0,T]}$, with respect to the path distribution, $Q^{\theta+\epsilon}_{[0,T]}$, has an explicit formula known also as Girsanov formula [8]

$$\frac{dQ^\theta_{[0,T]}}{dQ^{\theta+\epsilon}_{[0,T]}}(\{\mathbf{X}_t\}_{t=0}^T) = \frac{\nu^\theta(\mathbf{X}_0)}{\nu^{\theta+\epsilon}(\mathbf{X}_0)} \exp\left\{\int_0^T \sum_{j=1}^J \log \frac{a_j^\theta(\mathbf{X}_{t-})}{a_j^{\theta+\epsilon}(\mathbf{X}_{t-})}\, dN_t^j - \int_0^T [a_0^\theta(\mathbf{X}_t) - a_0^{\theta+\epsilon}(\mathbf{X}_t)]\, dt\right\} \ ,$$

where ν^{θ} (resp. $\nu^{\theta+\epsilon}$) is the initial distributions of $\{\mathbf{X}_t\}_{t\in\mathbb{R}_+}$ (resp. $\{\widetilde{\mathbf{X}}_t\}_{t\in\mathbb{R}_+}$) and the convention $\frac{0}{0}=1$ is assumed. Using the Girsanov formula, the relative entropy is rewritten as

$$\mathcal{R}\left(Q^{\theta}_{[0,T]}\,|\,Q^{\theta+\epsilon}_{[0,T]}\right) = \mathbb{E}_{Q^{\theta}_{[0,T]}}\left[\log\frac{\nu^{\theta}(\mathbf{X}_0)}{\nu^{\theta+\epsilon}(\mathbf{X}_0)} + \int_0^T\sum_{j=1}^J \log\frac{a_j^{\theta}(\mathbf{X}_{t-})}{a_j^{\theta+\epsilon}(\mathbf{X}_{t-})}\,dN_t^j - \int_0^T [a_0^{\theta}(\mathbf{X}_t) - a_0^{\theta+\epsilon}(\mathbf{X}_t)]\,dt\right]$$

$$= \mathbb{E}_{Q^{\theta}_{[0,T]}}\left[\log\frac{\nu^{\theta}(\mathbf{X}_0)}{\nu^{\theta+\epsilon}(\mathbf{X}_0)}\right] + \mathbb{E}_{Q^{\theta}_{[0,T]}}\left[\int_0^T\sum_{j=1}^J \log\frac{a_j^{\theta}(\mathbf{X}_{t-})}{a_j^{\theta+\epsilon}(\mathbf{X}_{t-})}\,dN_t^j\right] - \mathbb{E}_{Q^{\theta}_{[0,T]}}\left[\int_0^T [a_0^{\theta}(\mathbf{X}_t) - a_0^{\theta+\epsilon}(\mathbf{X}_t)]\,dt\right] .$$

Exploiting the fact that the process $M_t := N_t^j - \int_0^t a_j^{\theta}(\mathbf{X}_s)ds$ is a martingale, we have that

$$\mathbb{E}_{Q^{\theta}_{[0,T]}}\left[\int_0^T \log\frac{a_j^{\theta}(\mathbf{X}_{t-})}{a_j^{\theta+\epsilon}(\mathbf{X}_{t-})}\,dN_t^j\right] = \mathbb{E}_{Q^{\theta}_{[0,T]}}\left[\int_0^T \log\frac{a_j^{\theta}(\mathbf{X}_{t-})}{a_j^{\theta+\epsilon}(\mathbf{X}_{t-})}a_j^{\theta}(\mathbf{X}_{t-})\,dt\right] ,$$

for $j=1,\dots,J$. Moreover, changing the order of the integrals, the relative entropy is rewritten as

$$\mathcal{R}\left(Q^{\theta}_{[0,T]}\,|\,Q^{\theta+\epsilon}_{[0,T]}\right) = \mathcal{R}\left(\nu^{\theta}\,|\,\nu^{\theta+\epsilon}\right) + \int_0^T \mathbb{E}_{Q^{\theta}_{[0,T]}}\left[\sum_{j=1}^J a_j^{\theta}(\mathbf{X}_{t-})\log\frac{a_j^{\theta}(\mathbf{X}_{t-})}{a_j^{\theta+\epsilon}(\mathbf{X}_{t-})} - \left(a_0^{\theta}(\mathbf{X}_t) - a_0^{\theta+\epsilon}(\mathbf{X}_t)\right)\right] dt .$$

Following the discussion for the DTMC and slight abuse of notation, the pathwise relative entropy is written as

$$\mathcal{R}\left(Q^{\theta}_{[0,T]}\,|\,Q^{\theta+\epsilon}_{[0,T]}\right) = \mathcal{R}\left(\nu^{\theta}\,|\,\nu^{\theta+\epsilon}\right) + \int_0^T \mathcal{H}\left(Q_t^{\theta}\,|\,Q_t^{\theta+\epsilon}\right) dt , \tag{14}$$

where $\mathcal{H}\left(Q_t^{\theta}\,|\,Q_t^{\theta+\epsilon}\right) := \mathbb{E}_{Q^{\theta}_{[0,t]}}\left[\sum_{j=1}^J a_j^{\theta}(\mathbf{X}_{t-})\log\frac{a_j^{\theta}(\mathbf{X}_{t-})}{a_j^{\theta+\epsilon}(\mathbf{X}_{t-})} - \left(a_0^{\theta}(\mathbf{X}_t) - a_0^{\theta+\epsilon}(\mathbf{X}_t)\right)\right]$ is the instantaneous relative entropy. Finally, the pathwise FIM is obtained from a straightforward expansion of the relative entropy in terms of ϵ. It is given by

$$\mathcal{I}(Q^{\theta}_{[0,T]}) = \mathcal{I}(\nu^{\theta}) + \int_0^T \mathcal{I}_{\mathcal{H}}(Q_t^{\theta})\,dt , \tag{15}$$

where $\mathcal{I}(\nu^{\theta})$ is the FIM of the initial distribution, ν^{θ} while $\mathcal{I}_{\mathcal{H}}(Q_t^{\theta})$ can be thought as the instantaneous pathwise FIM given by

$$\mathcal{I}_{\mathcal{H}}(Q_t^{\theta}) = \mathbb{E}_{Q^{\theta}_{[0,t]}}\left[\sum_{j=1}^J a_j^{\theta}(\mathbf{X}_{t-})\nabla_{\theta}\log a^{\theta}(\mathbf{X}_{t-})\nabla_{\theta}\log a^{\theta}(\mathbf{X}_{t-})^T\right] . \tag{16}$$

3.1 Stationary regime

As already stated, the pathwise relative entropy at the stationary regime admits the decomposition given by (5). In the context of well-mixed reaction networks, the RER is explicitly written, [6],

$$\mathcal{H}\left(Q^{\theta}\,|\,Q^{\theta+\epsilon}\right) = \mathbb{E}_{\mu^{\theta}}\Big[\sum_{j=1}^J a_j^{\theta}(\mathbf{x})\log\frac{a_j^{\theta}(\mathbf{x})}{a_j^{\theta+\epsilon}(\mathbf{x})} - \left(a_0^{\theta}(\mathbf{x}) - a_0^{\theta+\epsilon}(\mathbf{x})\right)\Big] . \tag{17}$$

Finally, assuming smoothness of the propensity functions, $a_j^{\theta}(\mathbf{x})$, $j=1,\dots,J$, with respect to the parameter vector, θ, an FIM computed as the Hessian of RER is derived with explicit formula given by, [6],

$$\mathcal{I}_{\mathcal{H}}(Q^{\theta}) = \mathbb{E}_{\mu^{\theta}}\left[\sum_{j=1}^J a_j^{\theta}(\mathbf{x})\nabla_{\theta}\log a_j^{\theta}(\mathbf{x})\nabla_{\theta}\log a_j^{\theta}(\mathbf{x})^T\right] . \tag{18}$$

3.2 Integrated autocorrelation time for CTMC

Next, we derive the limit of the quantity $\tau_{\mu^\theta}^T(f) := \frac{1}{T}\mathrm{Var}_{\mu^\theta}(TF)$. By definition, we have that

$$\begin{aligned}
\tau_{\mu^\theta}^T(f) &= \frac{1}{T}\mathbb{E}_{Q^\theta_{[0,T]}}\big[\big(\int_0^T f(\mathbf{X}_t)dt - \mathbb{E}_{Q^\theta_{[0,T]}}[\int_0^T f(\mathbf{X}_t)dt]\big)^2\big] \\
&= \frac{1}{T}\int_{t=0}^T\int_{t=0}^T \mathbb{E}_{Q^\theta_{[0,T]}}[(f(\mathbf{X}_t) - \mathbb{E}_{Q^\theta_{[0,T]}}[f(\mathbf{X}_t)])(f(\mathbf{X}_s)] - \mathbb{E}_{Q^\theta_{[0,T]}}[f(\mathbf{X}_s))]dtds \\
&= \frac{1}{T}\int_{t=0}^T\int_{t=0}^T \mathbb{E}_{Q_{[s\wedge t, s\vee t]}^\theta}[(f(\mathbf{X}_t) - \mathbb{E}_{\mu^\theta}[f])(f(\mathbf{X}_s) - \mathbb{E}_{\mu^\theta}[f])]dtds \\
&= \frac{1}{T}\int_0^T\int_0^T < f(\mathbf{X}_t) - \mathbb{E}_{\mu^\theta}[f], f(\mathbf{X}_s) - \mathbb{E}_{\mu^\theta}[f] >_{\mu^\theta} dtds \ ,
\end{aligned}$$

where $< f(\mathbf{X}_t) - \mathbb{E}_{\mu^\theta}[f(\mathbf{x})], f(\mathbf{X}_s) - \mathbb{E}_{\mu^\theta}[f(\mathbf{x})] >_{\mu^\theta}$ is the stationary covariance between $f(\mathbf{X}_t)$ and $f(\mathbf{X}_s)$. Under a stationarity assumption, the covariance function depends only on the lag (i.e., the difference $|t-s|$), therefore, $\tau_{\mu^\theta}^T(f)$ can be simplified to

$$\tau_{\mu^\theta}^T(f) = \int_{-T}^T \big(1 - \frac{t}{T}\big) < f(\mathbf{X}_t) - \mathbb{E}_{\mu^\theta}[f], f(\mathbf{X}_0) - \mathbb{E}_{\mu^\theta}[f] >_{\mu^\theta} dt \ .$$

Sending $T \to \infty$, the continuous-time IAT is obtained:

$$\tau_{\mu^\theta}(f) = \lim_{T\to\infty}\tau_{\mu^\theta}^T(f) = \int_{-\infty}^\infty < f(\mathbf{X}_t) - \mathbb{E}_{\mu^\theta}[f], f(\mathbf{X}_0) - \mathbb{E}_{\mu^\theta}[f] >_{\mu^\theta} dt \ .$$

References

[1] T. Cover and J. Thomas. *Elements of Information Theory.* John Wiley & Sons, 1991.

[2] G. Casella and R.L. Berger. *Statistical Inference.* Duxbury advanced series in statistics and decision sciences. Thomson Learning, 2002.

[3] S. M. Kay. *Funtamentals of Statistical Signal Processing: Estimation Theory.* Prentice-Hall, Englewood Cliffs, NJ, 1993.

[4] P. Dupuis, M.A. Katsoulakis, Y. Pantazis, and P. Plecháč. Sesnitivity bounds and error estimates for stochastic models. *(in preparation).*

[5] Y. Pantazis and M. Katsoulakis. A relative entropy rate method for path space sensitivity analysis of stationary complex stochastic dynamics. *J. Chem. Phys.*, 138(5):054115, 2013.

[6] Y. Pantazis, M.A. Katsoulakis, and D. Vlachos. Parametric sensitivity analysis for biochemical reaction networks based on pathwise information theory. *BMC Bioinformatics*, 14(1):311, 2013.

[7] J. S. Liu. *Monte Carlo strategies in scientific computing.* Springer Series in Statistics. Springer-Verlag, New York, 2001.

[8] Pierre Bremaud. *Point Processes and Queues, Martingale Dynamics.* Springer-Verlag, 1981.
