# Supplementary material for: Accelerated Sensitivity Analysis in High-Dimensional Stochastic Reaction Networks
Source: PLoS One. 2015 Jul 10;10(7):e0130825. doi: 10.1371/journal.pone.0130825 (PMC4498611; doi:10.1371/journal.pone.0130825)
Supplement: S2 File — (PDF) [file pone.0130825.s002.pdf]

Unbiased Statistical Estimators for pathwise FIM and IAT

In both stationary and transient regimes, the numerical computation of the pathwise FIM as well as the variance of the time-averaged observable (IAT) should be performed. For the sake of completeness, we present the statistical estimators of these quantities which require simulations only from the unperturbed process, $\mathbf{X}_t$, i.e., only for the parameter value, θ. For both regimes, the unnormalized pathwise FIM can be written as (see S1 File)

$$\mathcal{I}(Q^{\theta}_{[0,T]}) = \mathbb{E}_{Q^{\theta}_{[0,T]}}\Big[\int_0^T \sum_{j=1}^J a_j^{\theta}(\mathbf{X}_{t-})\nabla_\theta \log a_j^{\theta}(\mathbf{X}_{t-})\nabla_\theta \log a_j^{\theta}(\mathbf{X}_{t-})^T dt\Big] \ ,$$

where we assume that both processes started from the same distribution, ν. Then, the n-th sample of the unbiased estimator for the pathwise FIM is given by [1, 2]

$$\begin{aligned}\bar{\mathcal{I}}^{(n)} = &\sum_{i=0}^{N_T^{(n)}-1} \delta t_i^{(n)} \sum_{j=1}^J a_j^{\theta}(\mathbf{x}_i^{(n)})\nabla_\theta \log a_j^{\theta}(\mathbf{x}_i^{(n)})\nabla_\theta \log a_j^{\theta}(\mathbf{x}_i^{(n)})^T \\ &+ \big(T - \sum_{i=0}^{N_T^{(n)}-1} \delta t_i^{(n)}\big) \sum_{j=1}^J a_j^{\theta}(\mathbf{x}^{(n)}_{N_T^{(n)}})\nabla_\theta \log a_j^{\theta}(\mathbf{x}^{(n)}_{N_T^{(n)}})\nabla_\theta \log a_j^{\theta}(\mathbf{x}^{(n)}_{N_T^{(n)}})^T \ .\end{aligned}$$

where $\delta t_i^{(n)}$ is an exponential random variable with parameter given by the total rate, $a_0^{\theta}(\mathbf{x}_i^{(n)})$, while $N_T^{(n)}$ is the number of jumps up to time T. The sequence $\{\mathbf{x}_i^{(n)}\}_{i=0}^{N_T^{(n)}}$ is the embedded Markov chain with transition probabilities from state $\mathbf{x}_i^{(n)}$ to state $\mathbf{x}_{i+1}^{(n)}$ given by the ratio $\frac{a_j^{\theta}(\mathbf{x}_i^{(n)})}{a_0^{\theta}(\mathbf{x}_i^{(n)})}$. The weight $\delta t_i^{(n)}$, which is the waiting time at state $\mathbf{x}_i^{(n)}$, is necessary for the unbiased estimation of the average value, [3]. Notice also that $\delta t_i^{(n)}$ can be replaced by its average which is the inverse of the total rate, $a_0^{\theta}(\mathbf{x}_i^{(n)})^{-1}$ Then, assuming that we simulate N trajectories, the unbiased estimator for the pathwise FIM is simply

$$\bar{\mathcal{I}} = \frac{1}{N}\sum_{n=1}^N \bar{\mathcal{I}}^{(n)} \ .$$

Along the same lines, unbiased estimators for the relative entropy and the relative entropy rate can be obtained, [1, 2].

Next, we discuss the computation of the variance of a time-averaged observable function. The unnormalized time-averaged observable for fixed T is given by

$$F = E_{Q^{\theta}_{[0,T]}}\Big[\int_{t=0}^T f(\mathbf{X}_t)dt\Big] \ .$$

The unbiased estimator of F for the n–th realization is

$$\bar{F}^{(n)} = \sum_{i=0}^{N_T^{(n)}-1} \delta t_i^{(n)} f(\mathbf{x}_i^{(n)}) + \big(T - \sum_{i=0}^{N_T^{(n)}-1} \delta t_i^{(n)}\big) f(\mathbf{x}_{N_T^{(n)}}) \ .$$

Then, the unbiased estimator of the mean of F is given by

$$\bar{F} = \frac{1}{N}\sum_{n=1}^{N} \bar{F}^{(n)} \ ,$$

while the unbiased estimator of the variance of F is given by

$$\bar{\sigma}_F^2 = \frac{1}{N-1}\sum_{n=1}^{N} \big(\bar{F}^{(n)} - \bar{F}\big)^2 \ .$$

We can obtain a statistical estimator for the sensitivity indices based on the coupling method (see File S3) along the same lines.

References

[1] Y. Pantazis and M. Katsoulakis. A relative entropy rate method for path space sensitivity analysis of stationary complex stochastic dynamics. *J. Chem. Phys.*, 138(5):054115, 2013.

[2] Y. Pantazis, M.A. Katsoulakis, and D. Vlachos. Parametric sensitivity analysis for biochemical reaction networks based on pathwise information theory. *BMC Bioinformatics*, 14(1):311, 2013.

[3] D. T. Gillespie. A general method for numerically simulating the stochastic time evolution of coupled chemical reactions. *J. Comp. Phys.*, 22:403–434, 1976.
