# Supplementary material for: Accelerated Sensitivity Analysis in High-Dimensional Stochastic Reaction Networks
Source: PLoS One. 2015 Jul 10;10(7):e0130825. doi: 10.1371/journal.pone.0130825 (PMC4498611; doi:10.1371/journal.pone.0130825)
Supplement: S3 File — A brief revision of the coupling method proposed by Anderson for the estimation of gradients in well-mixed reaction network systems. (PDF) [file pone.0130825.s003.pdf]

Coupling of Stochastic Processes

This section briefly revisits the coupling method proposed by Anderson [1] for the estimation of gradients in well-mixed reaction network systems. We recall that the sensitivity index of an observable of the stochastic process, $\mathbf{X}_t$, with respect to the parameter θ_k is defined as

$$S_{k,\ell} = \frac{\partial}{\partial \theta_k} \mathbb{E}_{Q^{\theta}_{[0,T]}} \left[F_\ell(\{\mathbf{X}_t\}_{t=0}^T) \right], \quad k = 1, \ldots, K \;\; \& \;\; \ell = 1, \ldots, L \; .$$

The partial derivative can be approximated by a second-order finite-difference scheme as

$$S_{k,\ell} \approx \tilde{S}_{k,\ell} = \frac{1}{2\epsilon_0} \Big(\mathbb{E}_{\mathbf{x}_0} \left[F_\ell(\{\mathbf{X}_t^{\theta_k+\epsilon_0}\}) \right] - \mathbb{E}_{\mathbf{x}_0} \left[F_\ell(\{\mathbf{X}_t^{\theta_k-\epsilon_0}\}) \right] \Big) \; , \tag{1}$$

where $\epsilon_0 \in \mathbb{R}$ and $\epsilon_0 \ll 1$. The expected value of the observable function $u(t, \mathbf{x}_0) := \mathbb{E}_{\mathbf{x}_0} F(\mathbf{X}_t)$ satisfies the initial value problem

$$\frac{d}{dt} u(t, \mathbf{x}_0) = \mathcal{L}^\theta u(t, \mathbf{x}_0), \quad u(0, \mathbf{x}_0) = F(\mathbf{x}_0)$$

where $\mathcal{L}^\theta$ is the generator operator defined by

$$\mathcal{L}^\theta F(\mathbf{x}) := \sum_{j=1}^J a_j^\theta(\mathbf{x}) \left[F(\mathbf{x} + \nu_j) - F(\mathbf{x}) \right] \; , \tag{2}$$

and $a_j^\theta(\cdot), j = 1, ..., J$, are the propensity functions.

In order to reduce the variance of the statistical estimator for $\tilde{S}_{k,\ell}$, we have to correlate the process $\mathbf{X}_t^{\theta_k+\epsilon_0}$ with the process $\mathbf{X}_t^{\theta_k-\epsilon_0}$. It has been shown in $[1, 2]$ that coupling indeed reduces the variance of the estimator, even in high-dimensional systems. In the coupling method, a new process is considered which is the pair of stochastic processes $\mathbf{Z}_t = (\mathbf{X}_t^{\theta_k+\epsilon_0}, \mathbf{X}_t^{\theta_k-\epsilon_0})$. We define the vector e_k which has zeros in all positions and 1 in the k–th position and set $\epsilon = \epsilon_0 e_k$. Then $\mathbf{Z}_t$ is a Markov process with infinitesimal generator,

$$\begin{aligned}
\bar{\mathcal{L}} G(\mathbf{z}) = & \sum_{j=1}^J \min\{a_j^{\theta+\epsilon}(\mathbf{x}), a_j^{\theta-\epsilon}(\mathbf{y})\} \left[G(\mathbf{x} + \nu_j, \mathbf{y} + \nu_j) - G(\mathbf{x}, \mathbf{y}) \right] \\
& + \sum_{j=1}^J \left[a_j^{\theta+\epsilon}(\mathbf{x}) - \min\{a_j^{\theta+\epsilon}(\mathbf{x}), a_j^{\theta-\epsilon}(\mathbf{y})\} \right] \left[G(\mathbf{x} + \nu_j, \mathbf{y}) - G(\mathbf{x}, \mathbf{y}) \right] \\
& + \sum_{j=1}^J \left[a_j^{\theta-\epsilon}(\mathbf{y}) - \min\{a_j^{\theta+\epsilon}(\mathbf{x}), a_j^{\theta-\epsilon}(\mathbf{y})\} \right] \left[G(\mathbf{x}, \mathbf{y} + \nu_j) - G(\mathbf{x}, \mathbf{y}) \right],
\end{aligned} \tag{3}$$

where $\mathbf{z} = (\mathbf{x}, \mathbf{y})$ is the state of the $\mathbf{Z}_t$ process and G an observable function on $\mathbf{Z}_t$. The transitions of the coupled generator are shown in Figure 1. More specifically, the intuition behind this coupling is that both processes move to the same updating direction with the minimum rate $c_j(\mathbf{x}, \mathbf{y}) = \min\{a_j^{\theta+\epsilon}(\mathbf{x}), a_j^{\theta-\epsilon}(\mathbf{y})\}$

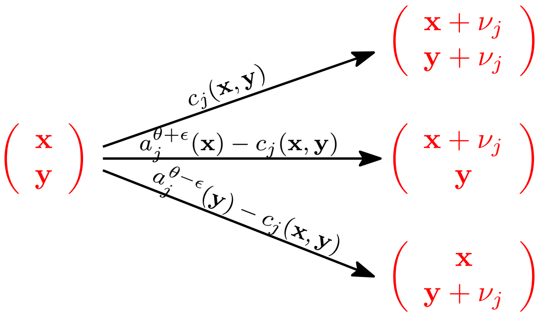

Figure 1: Transitions of the coupled process $\mathbf{z} = (\mathbf{x}, \mathbf{y})$ where $c_j(\mathbf{x}, \mathbf{y}) = \min\{a_j^{\theta+\epsilon}(\mathbf{x}), a_j^{\theta-\epsilon}(\mathbf{y})\}$ for $j = 1, \ldots, J$.

for $j = 1, \ldots, J$, leading to trajectories that most of the time track each other. On the other hand, every process has to do some independent jumps in order to keep the correct dynamics. Indeed, it can be proved, [3], that the coupled generator (3) satisfies

$$\mathbb{E}_{\mathbf{z}_0}\left[G(\mathbf{X}_t^{\theta+\epsilon}, \mathbf{X}_t^{\theta-\epsilon})\right] = \mathbb{E}_{\mathbf{x}_0}\left[F(\mathbf{X}_t^{\theta+\epsilon})\right] \text{ when } G(\mathbf{x}, \mathbf{y}) = F(\mathbf{x}) \text{ and}$$
$$\mathbb{E}_{\mathbf{z}_0}\left[G(\mathbf{X}_t^{\theta+\epsilon}, \mathbf{X}_t^{\theta-\epsilon})\right] = \mathbb{E}_{\mathbf{y}_0}\left[F(\mathbf{X}_t^{\theta-\epsilon})\right] \text{ when } G(\mathbf{x}, \mathbf{y}) = F(\mathbf{y}),$$

where $\mathbb{E}_{\mathbf{z}_0}\left[G(\mathbf{X}_t^{\theta+\epsilon}, \mathbf{X}_t^{\theta-\epsilon})\right]$ denotes the mean value of the observable function G of the state variables $(\mathbf{X}_t^{\theta+\epsilon}, \mathbf{X}_t^{\theta-\epsilon})$ with respect to the law imposed by the initial data $\mathbf{z}_0 = (\mathbf{x}_0, \mathbf{y}_0)$. These relations imply that all averages of the two coupled processes, and thus all observables F, coincide with the averaged observables generated by the uncoupled generators $\mathcal{L}^{\theta+\epsilon}, \mathcal{L}^{\theta-\epsilon}$.

References

[1] David F. Anderson. An efficient finite difference method for parameter sensitivities of continuous-time Markov chains. *SIAM J. Numerical Analysis*, 50(5):2237–2258, 2012.

[2] Georgios Arampatzis and Markos Katsoulakis. Goal-oriented sensitivity analysis for lattice kinetic monte carlo simulations. *J. Chem. Phys.*, 12(140):124108, 2014.

[3] Thomas M. Liggett. *Interacting Particle Systems*, volume 276 of *Grundlehren der mathematischen Wissenschaften*. Springer-Verlag, New York, Berlin, Heidelberg, Tokyo, 1985.
