# Supplementary material for: Accelerated Sensitivity Analysis in High-Dimensional Stochastic Reaction Networks
Source: PLoS One. 2015 Jul 10;10(7):e0130825. doi: 10.1371/journal.pone.0130825 (PMC4498611; doi:10.1371/journal.pone.0130825)
Supplement: S4 File — An approximation of the variance of the SB is first presented. Then, we present the technical details for the computational cost comparison of the proposed strategy with the coupling method applied to all the SIs of the model. (PDF) [file pone.0130825.s004.pdf]

Computation Cost based on Variance Estimates

In the first section of this supporting information file, we derive an approximation of the variance of the upper bound inequality ((4) in Main Text) by approximating the variance of the square root of the product of two dependent random variables, see Lemma 1.2. In the second section, we present the technical details for the comparison of the proposed strategy with the coupling method applied to all the sensitivity indices (SIs) of the model, discussed in section "Computational Cost" in the Main Text.

1 Variance of the estimator of the sensitivity bound

In order to get confidence intervals for an estimator that consists of simpler estimators, e.g., the estimator of the sensitivity bound ((4) in Main Text), the variance of the complex estimator must be computed. The Delta method gives a way to express the variance of the complex estimator using the variances of the simpler ones. In the following discussion we denote by $\mathcal{N}(0,\Sigma)$ the normal distribution with mean 0 and covariance matrix Σ and by $\xrightarrow{d}$ the convergence in distribution.

Theorem 1.1 (Delta method)**.** *Suppose $\{X_n\}_{n=0}^{\infty}$ is a sequence of random vectors in $\mathbb{R}^K$ such that*

$$\sqrt{n}\,(X_n-\theta) \;\xrightarrow{d}\; \mathcal{N}(0,\Sigma), \tag{1}$$

for some $\theta\in\mathbb{R}^K$ and $\Sigma\in\mathbb{R}^{K\times K}$. Let $g:\mathbb{R}^K\to\mathbb{R}$ and $J(\theta)=\nabla g(x)|_{x=\theta}$. Then,

$$\sqrt{n}\big(g(X_n)-g(\theta)\big) \;\xrightarrow{d}\; \mathcal{N}\big(0, J(\theta)^T\Sigma J(\theta)\big). \tag{2}$$

Proof. See Theorem 3.1 in [1]. □

The practical interpretation of the Delta method is that for large fixed N the variance of $g(X_N)$ can be approximated by $\frac{1}{N}J(\theta)^T\Sigma J(\theta)$. After a Definition and an intermediate Lemma we will prove a Lemma that gives and approximation for the variance of the sensitivity bound ((4) in Main Text).

Definition 1.1 (moments)**.** *For a random variable X we define the moments,*

$$\mu_k=\begin{cases}\mathbb{E}[X], & k=1\\ \mathbb{E}\left[(X-\mathbb{E}[X])^k\right], & k>1\,,\end{cases} \tag{3}$$

and the central moments

$$m_k=\mathbb{E}\left[X^k\right], \quad k=1,2,\dots\,. \tag{4}$$

Moreover, for two random variables, X and Y, we define the joint moments by,

$$\xi_{k,\ell}=\mathbb{E}\left[(X-\mathbb{E}[X])^k(Y-\mathbb{E}[Y])^\ell\right], \quad k,\ell=1,2,\dots\,. \tag{5}$$

Lemma 1.1. *Assume $\{X_i\}_{i=1}^N$ are independent and identically distributed random variables. Then the following are true:*

1. an unbiased estimator for the mean, μ_1, of distribution f is given by

$$\overline{X} = \frac{1}{N}\sum_{i=1}^{N} X_i\,, \tag{6}$$

and holds that

$$\sqrt{N}\left(\overline{X} - \mu_1\right) \xrightarrow{d} \mathcal{N}(0, \mu_2)\,, \tag{7}$$

2. an unbiased estimator for the variance, μ_2, of distribution f is given by

$$S^2 = \frac{1}{N-1}\sum_{i=1}^{N}\left(X_i - \overline{X}\right)^2\,, \tag{8}$$

with variance equal to

$$\sqrt{N}\left(S^2 - \mu_2\right) \xrightarrow{d} \mathcal{N}(0, \mu_4 - \mu_2^2)\,. \tag{9}$$

Proof. The proof of the first part is an application of the Central Limit Theorem and can be found in [1]. The second part can be proved using the Delta method and the details can be found in Section 3.1 of [1]. □

Lemma 1.2. *Let $\{X_i\}_{i=1}^N$ and $\{Y_i\}_{i=1}^N$ be independent and identically distributed random variables. We assume that the central moments μ_1 of X and $\nu_1, \nu_2, \nu_3, \nu_4$ of Y are finite and in particular $\mu_1, \nu_2 \neq 0$. Moreover, the joint moment $\xi_{1,2}$ is finite.*

Let $\overline{X}$ be an estimator for $\mathbb{E}[X_1]$ and S_Y^2 an estimator for $\mathrm{Var}[Y_1]$, *as defined in Lemma 1.1. Then, it holds that*

$$\sqrt{N}\left(\sqrt{\overline{X}\,S_Y^2} - \sqrt{\mu_1\,\nu_2}\right) \xrightarrow{d} \mathcal{N}(0, \lambda)\,, \tag{10}$$

where

$$\lambda = \frac{\nu_2}{4\mu_1}\mu_2 + \frac{\mu_1}{4\nu_2}\left(\nu_4 - \nu_2^2\right) + \frac{1}{2}\xi_{1,2}\,. \tag{11}$$

Proof. For simplicity in the presentation we will work with the estimator $S^2 = \frac{1}{N}\sum_{i=1}^{N}\left(Y_i - \overline{Y}\right)^2$ instead of (8). The same result holds for the estimator (8), see the Example 3.2 in [1].

First, notice that $\sqrt{\overline{X}\,S_Y^2}$ is equal to $\phi(\overline{X}, \overline{Y}, \overline{Y^2})$ for $\varphi(x, y, z) = \sqrt{x(y^2 - z)}$. Moreover, from the multivariate Central Limit Theorem, see [1], it holds that,

$$\sqrt{N}\begin{pmatrix}\overline{X}\\ \overline{Y}\\ \overline{Y^2}\end{pmatrix} - \sqrt{N}\begin{pmatrix}m_1\\ n_1\\ n_2\end{pmatrix} \xrightarrow{d} \mathcal{N}(0, \Sigma)\,, \tag{12}$$

where the covariance matrix Σ is equal to

$$\Sigma = \mathbb{E}\Big[\left(X - m_1, Y - n_1, Y^2 - n_2\right)\left(X - m_1, Y - n_1, Y^2 - n_2\right)^T\Big]\,. \tag{13}$$

The Jacobian of φ evaluated at $\theta = (m_1, n_1, n_2)$ is equal to

$$J(\theta) = \frac{1}{2\sqrt{\mu_1\nu_2}}\left(\nu_2,\, 2\mu_1\nu_1,\, -\mu_1\right)^T\,. \tag{14}$$

Finally, use Theorem 1.1 to conclude equation (10) with λ given by

$$\lambda = J(\theta)^T\,\Sigma\,J(\theta)\,. \tag{15}$$

Equation (11) follows after basic algebraic manipulations. □

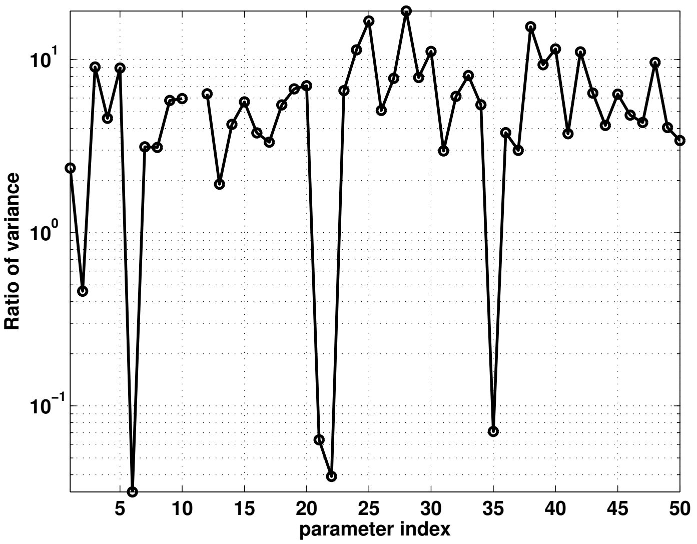

Figure A: The ratio of the variance of the coupling estimator ((12) in Main Text) to the variance of the bound estimator ((4) in Main Text) is presented here for all parameters of the EGFR model. The sum of these values contribute to the G_1 term in formula (22). This result indicates that the variance of the bound estimator is much less than that of the coupling estimator. The ratio of variance for Parameter 11 does not appear in the graph because the variance of the coupling estimator is equal to zero.

2 Technical details regarding the computational cost

In this section, we compare the computational cost of (a) the proposed strategy, involving estimation of the upper bound in the inequality ((4) in Main Text) and the subsequent estimation of just the most significant sensitivity indices (SIs), $\tilde{S}_{k,\ell}$m (see (12) in Main Text), to (b) the estimation of the full sensitivity matrix, using the finite-difference coupling method for each one of the entries of the sensitivity matrix. The comparison will be done under the requirement that the *relative confidence intervals* of all estimators involved in both approaches will be of length equal to $\delta \ll 1$. The relative confidence interval is defined as the usual confidence interval, however the estimator of the standard deviation is replaced by a normalized standard deviation

$$\hat{\hat{\sigma}} = \begin{cases} \frac{\hat{\sigma}}{|\overline{x}|}, & |\overline{x}| > \epsilon \\ \hat{\sigma}, & \text{otherwise.} \end{cases} \tag{16}$$

where $\hat{\sigma}$ is the estimated standard deviation of the random variable X and $\overline{x} = \frac{1}{N}\sum_{i=1}^{N} x_i$ is the estimator of the mean of X. With this definition, random variables with large mean values have standard deviation comparable with random variables with small mean values. ϵ plays the role of a separator between large and small values. In our study we used $\epsilon = 0.01$.

Next, we set notation before proceeding to the comparison of the estimators. The time averaged observable will be that of equation (1) in Main Text with $T = 10$ and $f_\ell(\mathbf{x}) = x_\ell,\ \ell = 1, \ldots, N$, for the particular model considered here. The length of the relative confidence interval for the coupling estimator ((12) in Main Text) for the k–th parameter and the ℓ–th observable, using $M_{k,\ell}$ samples, is equal to

$$L^c_{k,\ell} = 2\alpha \frac{V^c_{k,\ell}}{\sqrt{M_{k,\ell}}},\ \ k = 1, \ldots, K,\ \ \ell = 1, \ldots, N, \tag{17}$$

where $V^c_{k,\ell}$ is the estimated normalized standard deviation, as defined in (16), using $M_{k,\ell}$ samples, and $\alpha = 1.96$ is the inverse of the cumulative normal distribution evaluated at 0.975 leading to a 95% confidence interval, see [2]. The superscript c in (17) stands for the coupling method, see Section "Step 2: Finding the most sensitive parameters" in Main Text. Under the requirement that $L^c_{k,\ell} = \delta$ we get the minimum number of samples for every pair (k, ℓ)

$$M^c_{k,\ell} = \left\lceil \left(\frac{2\alpha}{\delta} V^c_{k,\ell} \right)^2 \right\rceil, \tag{18}$$

where $\lceil x \rceil$ is the ceiling function defined by $\lceil x \rceil = \min\{n \in \mathbb{Z} : \; n \geq x\}$. In the coupling method, as a variant of the SSA method [3], for every perturbed parameter we compute the SIs of all species. Hence, the minimum number of trajectories needed to be produced by this method is determined by the species with the highest variance. Thus, we define the quantity

$$M^c_k = \max_{1 \leq \ell \leq N} M^c_{k,\ell}. \tag{19}$$

Let M^c be the sum of M^c_k over all k, i.e. the total number of samples required by the coupling method such that all estimated SIs will have a relative confidence interval equal to δ. This number will be compared to the minimum samples required by the method proposed in this paper under the requirement that all estimators' relative confidence interval will be of the same length.

Working in the same way, we find the minimum number of trajectories required in order the relative confidence interval of the estimator of the sensitivity bound (SB) ((4) in Main Text) to be less or equal to δ,

$$M^b_{k,\ell} = \left\lceil \left(\frac{2\alpha}{\delta} V^b_{k,\ell} \right)^2 \right\rceil, \tag{20}$$

where the superscript b stands for "bound". The statistical estimators of the SB are presented in supporting information File S2 and the variance of the estimator is computed using Lemma 1.2. In one trajectory we collect samples from both FIM and IAT, i.e. the minimum number of iterations will be determined by the bound of the sensitivity of the ℓ–th species of the k–th parameter with the higher variance. Thus we define the quantity

$$M^b = \max_{\substack{1 \leq k \leq K \\ 1 \leq \ell \leq N}} M^b_{k,\ell}, \tag{21}$$

which is the minimum number of samples required for the upper bound estimator to have a relative confidence interval of less or equal to δ.

In order to compare the straightforward SI estimator ((12) in Main Text) with the strategy proposed here, we have to compare the numbers in (19) and (21). Note that in our strategy, after the first screening using the SB is performed, we have to estimate accurately the SIs using the coupling method. Thus the ratio of the total computational cost of the proposed strategy to the computational cost of coupling in terms of number of samples is,

$$G = \frac{M^b + \sum_{k \in S} M^c_k}{\sum_{k=1}^K M^c_k} = \frac{1}{1 + \sum_{k=1}^K \frac{M^c_k}{M^b}} + \frac{1}{1 + \frac{\sum_{k \in I} M^c_k}{\sum_{k \in S} M^c_k}} = G_1 + G_2 \tag{22}$$

where I and S are the sets of indices of insensitive and sensitive parameters, respectively. Note that in the above equation we dropped the ceiling functions in (19) and (21) making the calculation easier and introducing a negligible error. The interpretation of G_1 is *"how many times is the coupling faster than Step 1 of the proposed strategy"*, i.e. the computation of the upper bound, and that of G_2 *"how many times is the coupling applied to all sensitivity indices is faster than Step 2 of the proposed strategy"*, i.e. neglecting SIs with small values. The ratio $1/G$ is the total speed-up of the proposed two–step strategy.

We argue that the estimation of the SB is much faster than that of the actual SIs using the coupling method for the same confidence interval and the same ϵ in the normalized standard deviation (16). This means that $G_1 \ll 1$ and the important term in (22) is G_2. In Figure 1, the ratio

$$G_{1,k} = \frac{M_k^c}{M^b}, \quad k = 1, \ldots, K \tag{23}$$

is plotted in logarithmic scale for the EGFR model described in Section "The EGFR model" in Main Text with $\epsilon = 0.01$ in definition (16). The G_1 term is equal to 0.0034 showing that the estimation of the SB needs about 300 times less samples than that of the computation of SIs using the coupling method. Finally, we can get an approximation to G_2 by arguing that M_k^c is approximately the same for all parameters, $k = 1, \ldots, K$. Then, from (22), $G_2 \approx \frac{K'}{K}$, where K' is the size of the set S, i.e., K' is the number of sensitive parameters. Under the assumption that $G_1 \ll 1$ we get an approximation for the total computational gain,

$$G \approx \frac{K'}{K}. \tag{24}$$

References

1. A. W. van der Vaart. *Asymptotic Statistics.* Cambridge University Press, 1998.
2. L. Wasserman. *All of Statistics: A Concise Course in Statistical Inference.* Springer, 2004.
3. D. T. Gillespie. A general method for numerically simulating the stochastic time evolution of coupled chemical reactions. *J. Comp. Phys.*, 22:403–434, 1976.
